# The genotype–phenotype correlations of the *CACNA1A*-related neurodevelopmental disorders: a small case series and literature reviews

**DOI:** 10.3389/fnmol.2023.1222321

**Published:** 2023-07-24

**Authors:** Miriam Kessi, Baiyu Chen, Nan Pang, Lifen Yang, Jing Peng, Fang He, Fei Yin

**Affiliations:** ^1^Department of Pediatrics, Xiangya Hospital, Central South University, Changsha, China; ^2^Hunan Intellectual and Developmental Disabilities Research Center, Pediatrics, Changsha, China; ^3^Clinical Research Center for Children’s Neurodevelopmental Disabilities of Hunan Province, Xiangya Hospital, Central South University, Changsha, China

**Keywords:** *CACNA1A*, genotype–phenotype correlations, global developmental delay, intellectual disability, epilepsy, autism spectrum disorder

## Abstract

**Background:**

Genotype–phenotype correlations of the *CACNA1A*-related neurodevelopmental disorders such as global developmental delay (GDD)/intellectual disability (ID), epileptic encephalopathy (EE), and autism spectrum disorder (ASD) are unknown. We aimed to summarize genotype–phenotype correlations and potential treatment for *CACNA1A*-related neurodevelopmental disorders.

**Methods:**

Six children diagnosed with *CACNA1A*-related neurodevelopmental disorders at Xiangya Hospital, Central South University from April 2018 to July 2021 were enrolled. The PubMed database was systematically searched for all reported patients with *CACNA1A*-related neurodevelopmental disorders until February 2023. Thereafter, we divided patients into several groups for comparison.

**Results:**

Six patients were recruited from our hospital. Three cases presented with epilepsy, five with GDD/ID, five with ataxia, and two with ASD. The variants included p.G701R, p.R279C, p.D1644N, p.Y62C, p.L1422Sfs*8, and p. R1664Q [two gain-of-function (GOF) and four loss-of-function (LOF) variants]. About 187 individuals with GDD/ID harboring 123 variants were found (case series plus data from literature). Of those 123 variants, p.A713T and p.R1664* were recurrent, 37 were LOF, and 7 were GOF. GOF variants were linked with severe-profound GDD/ID while LOF variants were associated with mild–moderate GDD/ID (*p* = 0.001). The p.A713T variant correlated with severe-profound GDD/ID (*p* = 0.003). A total of 130 epileptic patients harboring 83 variants were identified. The epileptic manifestations included status epilepticus (*n* = 64), provoked seizures (*n* = 49), focal seizures (*n* = 37), EE (*n* = 29), absence seizures (*n* = 26), and myoclonic seizures (*n* = 10). About 49 (42.20%) patients had controlled seizures while 67 (57.80%) individuals remained with refractory seizures. Status epilepticus correlated with variants located on S4, S5, and S6 (*p* = 0.000). Among the 83 epilepsy-related variants, 23 were recurrent, 32 were LOF, and 11 were GOF. Status epilepticus was linked with GOF variants (*p* = 0.000). LOF variants were associated with absence seizures (*p* = 0.000). Six patients died at an early age (3 months to ≤5 years). We found 18 children with ASD. Thirteen variants including recurrent ones were identified in those 18 cases. GOF changes were more linked to ASD.

**Conclusion:**

The p.A713T variant is linked with severe-profound GDD/ID. More than half of *CACNA1A*-related epilepsy is refractory. The most common epileptic manifestation is status epilepticus, which correlates with variants located on S4, S5, and S6.

## Introduction

*CACNA1A* (calcium voltage-gated channel subunit alpha1 A) is a bicistronic gene that encodes both the alpha-1 (α1A) subunit of a calcium channel, also called Cav2.1 (Bourinet et al., 1999), and α1ACT (a transcription factor). Cav2.1 channels are highly expressed in the cerebral cortex, hippocampus, and cerebellum (Kessi et al., 2021). They are found in the pre-synaptic area, where they stimulate the secretion of neurotransmitters which hasten synaptic transmission as well as synapse plasticity (Martínez-Monseny et al., 2021). *CACNA1A* variants are related to a broad spectrum of neurological phenotypes. The classical phenotypes include episodic ataxia 2 (EA2) which is mostly caused by nonsense mutations (Strupp et al., 2007), familial hemiplegic migraine type 1 (FHM1) which is frequently caused by missense mutations (mainly gain-of-function (GOF) variants) (Ducros et al., 2001), and spinocerebellar ataxia type 6 (SCA6) which usually occurs because of the expanded CAG repeats (Klockgether, 2008). However, with advanced next-generation sequencing techniques, *CACNA1A* variants have been linked to more wider phenotypic spectrum including global developmental delay (GDD)/intellectual disability (ID), epileptic encephalopathy (EE), and autism spectrum disorder (ASD) (Kessi et al., 2021; Indelicato and Boesch, 2023). Currently, genotype–phenotype correlations of the *CACNA1A* – related neurodevelopmental disorders such as GDD/ID, ASD, and epilepsy are unknown.

Thus, we aimed to investigate genotype–phenotype correlations, underlying mechanisms, and potential therapies for the for *CACNA1A*-related neurodevelopmental disorders. Knowledge about genotype–phenotype correlations will aid in knowing disease prognosis which might help in the preparation of future research.

## Materials and methods

### Ethical considerations

The institutional ethics committee of Xiangya Hospital, Central South University reviewed and approved this study according to the World Medical Association on ethical principles of human research for medical purposes agreement. Written informed consent was acquired from the parents or guardians of the subjects.

### Human subjects

Children diagnosed with *CACNA1A*-related neurodevelopmental disorders in the pediatric neurology department, Xiangya Hospital, Central South University from April 2018 to July 2021 were recruited. In addition, we collected information of all reported patients with *CACNA1A*-related neurodevelopmental disorders systematically from the PubMed database. The keywords used for searching articles were the combination of the *CACNA1A* OR Cav2.1 channel AND (epilepsy OR intellectual disability OR global developmental delay OR autism spectrum disorder OR neurological disease OR neurological disorder OR treatment OR animal model OR mechanisms) for all years to date February 2023. This review included only clinical papers published in English, however, the discussion was supplemented by all animal or mechanisms studies available in the literature. It excluded abstracts, patents, reviews, book chapters, and conference papers. The final reference list was produced according to the originality and relevance to the broad scope of this study. Overall, 1024 papers were found at the first search which were filtered to get the desired information. About 90 papers that met the inclusion criteria were included in the final analysis (Supplementary Flow Chart 1). The systematic review was carried out based on the PRISMA 2020 statement: an updated guideline for reporting systematic reviews (Page et al., 2021).

### Study design and grouping of the patients

The cross-sectional retrospective cohort study was conducted. This was accompanied by a comprehensive literature review of all *CACNA1A* reported cases in the literature. Our patients along with those reported in the literature were divided into groups according to the major phenotypes; epilepsy, GDD/ID, and ASD. We further categorized patients into other smaller groups for comparison, particularly for the epilepsy and GDD/ID groups. For ID/GDD patients, we created groups of patients with GOF and loss of function (LOF) variants, individuals with missense and nonsense variants as well as patients with mild–moderate and severe-profound ID/GDD. For the epileptic patients, we formed groups of patients with GOF and LOF variants, individuals with missense and nonsense variants, patients with status epilepticus, absence, and myoclonic seizures as well as patients with controlled seizures (including those who achieved seizure freedom), and refractory epilepsy. The sample size depended on the available *CACNA1A*-related disordered patients from our hospital and in the literature.

For the case series, epilepsy was diagnosed according to International League against Epilepsy (ILAE) standards. Whereas, GDD/ID and ASD were diagnosed according to diagnostic criteria of the DSM-5 for Intellectual disabilities (Diagnostic and Statistical Manual of Mental Disorders, Fifth Edition, American Psychiatric Association 2013). Clinical interviews, observations, and standardized age-related rating scales were used for assessment of the adaptive functioning. The GDD diagnosis was often initially formulated based on clinical judgment rather than on formal standardized assessments, especially for young patients (Shevell, 2008). We used standardized age-related rating scales such as Gesell Developmental Schedules for patients younger than 2–4 years of age, Wechsler Preschool and Primary Scale of Intelligence Fourth Edition (WPPSI-IV) for patients between 4 and 6 years, and Wechsler Intelligence Scale for Children, Fourth Edition (WISC-IV) for patients who were 6 years old or older than 6 years. The GDD/ID severity was graded as described in our previously published articles (Kessi et al., 2018, 2023). For the cases from the literature, there was no specific definition for GDD/ID. Some of the authors defined GDD/ID according to DSM-5 and provided information in regards to severity while other authors did not provide the severity grades. The information of all GDD/ID patients was collected, however, we only analyzed those with information related to GDD/ID severity.

### Data content and collection methods

Data was collected by senior neurologists and medical geneticists. The expert opinions of radiologists and electroencephalogram technicians were considered. Clinical characteristics of the patients such as demographic data, prenatal, natal, post-natal, family and behavioral histories, seizure characteristics, growth and development assessment results, and neurological examinations were collected. Auxiliary examination findings including blood and urine test results, brain magnetic resonance imaging (MRI) or computerized tomography (CT) results, cardiac and kidney imaging findings, electroencephalography (EEG) findings, metabolic findings, and genetic test results along with their functional experimental results were collected.

### Sequencing, mutations analysis and interpretation

After informing and receiving the signed consent forms from the patients, guardians, or parents, blood samples of the patients and their biological parents were collected. Genomic DNA was extracted, prepared, and analyzed according to the previous protocols. Whole exome sequencing (WES) was performed on all six cases. Sanger sequencing confirmed the parenteral origin of the variants. The genetic results were interpreted as per American College of Medical Genetics (ACMG) guidelines (Richards et al., 2015; Riggs et al., 2020).

### Statistical analysis

Statistical analysis was performed by using the Statistical Package for Social Science (IBM, SPSS Statistics Version 25). Categorical data were summarized in the form of frequencies and proportions, and analyzed with the Chi-square test. Mann–Whitney test was used to compare non-parametric continuous data. The *p* ≤ 0.05 indicated significant differences between groups.

## Results

### Clinical features of the patients recruited from our hospital

A total of six cases were recruited from our hospital: three boys and three girls. The onset age ranged from 1 year and 20 days to 10 years. Three cases presented with seizures (three focal and one absence seizures, and one had status epilepticus). Two cases had a previous history of febrile seizures. Five cases had GDD/ID, five cases had ataxia, and two had ASD. Two cases had profound GDD/ID, one severe and two mild. Three cases had cerebellar atrophy (Supplementary Table 1). We identified six *CACNA1A* variants of which four were *de novo* and two inherited. The variants included p.G701R, p.R279C, p.D1644N, p.Y62C, p.L1422Sfs*8, and p. R1664Q (2 GOF and 4 LOF variants) (Table 1). These variants can be found in ClinVar (SCV003930352 – SCV003930359).

**Table 1:** Genetic summary of 6 patients with *CACNA1A*-associated neurodevelopmental disorders from our hospital.

	P1	P2	P3	P4	P5	P6
Chromosome position	chr19:13414587	chr19:13470563	chr19:13356019	chr19:13616854	chr19:13370505	Chr19:13235693
Nucleic acid alteration	c.2101G > A	c.835C > T	c.4930G > A	c.185A > G	c.4264delC	c.4991G > A
Amino acid change	p. G701R	p. R279C	p. D1644N	p. Y62C	p. L1422Sfs*8	p. R1664Q
Transcript	NM_023035	NM_023035	NM_001127221.1	NM_001127221	NM_001127221.1	NM_001127221.2
Exon/intron	Exon16	Exon 6	Exon 31	Exon 1	Exon 27	Exon 32
Structural domain	II	I	IV	I	IV	IV
Heterozygosity	Heterozygous	Heterozygous	Heterozygous	Heterozygous	Heterozygous	Heterozygous
Genetic pattern	AD	AD	AD	AD	AD	AD
Location	S6 domain II	Extracellular	S3 domain IV	Cytoplasmic	Extracellular	S4 domain IV
P1	P2	P3	P4	P5	P6	P1
Mode of inheritance	*De novo*	Inherited from the father	*De novo*	*De novo*	Inherited from the mother	*De novo*
Mutation taster	Disease causing	Disease causing	Disease causing	Disease causing	Disease causing	Disease causing
POLYPHEN	Probably damaging (score: 1.000)	Possibly damaging (score: 0.939)	Probably damaging (score: 1.000)	Probably damaging (score: 0.999)	–	Probably damaging (score: 1.000)
PROVEAN PROTEIN	–	Deleterious (score: −5.780)	–	Deleterious (score: −6.542)	–	–
ACMG score	LP	P	LP	LP	LP	P
Functional effect	LOF	LOF	GOF	GOF	LOF	LOF
Final classification	P	P	P	P	P	P

ACMG, American College of Medical Genetics; AD, autosomal dominant; P, pathogenic; LP, likely pathogenic; LOF, loss-of-function; GOF, gain-of-function.

### Epilepsy

We collected information on patients with epilepsy from our hospital and combined them with those reported in the literature. We found 130 patients carrying 83 pathogenic or likely pathogenic *CACNA1A* variants. Of the 83 variants, 23 were recurrent.

#### General information about our patients and those reported in the literature

Ninety-two individuals had information related to sex, of whom 45 were males and 47 were females. The mean age of seizure onset was 59 months, the median age was 25 months ± 107.5SD, and the age range was 0 days – 50 years. Of the 130 patients, there were 42 (32.30%) from China, 27 (20.76%) from the USA, 18 (13.84%) from France and Belgium, 18 (13.85%) from Korea, 6 (4.62%) from the UK, 6 (4.62%) from the Netherlands, 4 (3.078%) from ‘North America’, 4 (3.08%) from Italy, 3 (2.31%) from Iran, 3 (2.31%) from Canada, 3 (2.31%) from Japan, 3 (2.31%) from Estonia, 2 (1.54%) from Germany, 1 (0.77%) from Poland, 1 (0.77%) from India, 1 (0.77%) from Australia, and it was unclear which country 5 (3.85%) were from (from the Epilepsy Phenome/Genome Project). Of the 116 patients with information about the treatment outcome, 67 (57.80%) individuals remained with refractory seizures while 49 (42.20%) achieved seizure control. Six cases died at a very young age (from the age of 3 months – ≤ 5 years) (Supplementary Table 2). The epileptic phenotypes were very diverse and had some provoking factors for the 49 cases. The epileptic manifestations included status epilepticus (*n* = 64), provoked seizures (*n* = 49), focal seizures (*n* = 37), epileptic encephalopathy (EE, *n* = 29), absence seizures (*n* = 26), and myoclonic seizures (*n* = 10) (Supplementary Figure 1). Seizure-provoking factors included fever for most of the cases, infection, mild head trauma, stress, excitation, bathing, climatic changes, excitation, agitation, and traveling.

#### Genotypes of the *CACNA1A* related epilepsy

**Figure 1:**
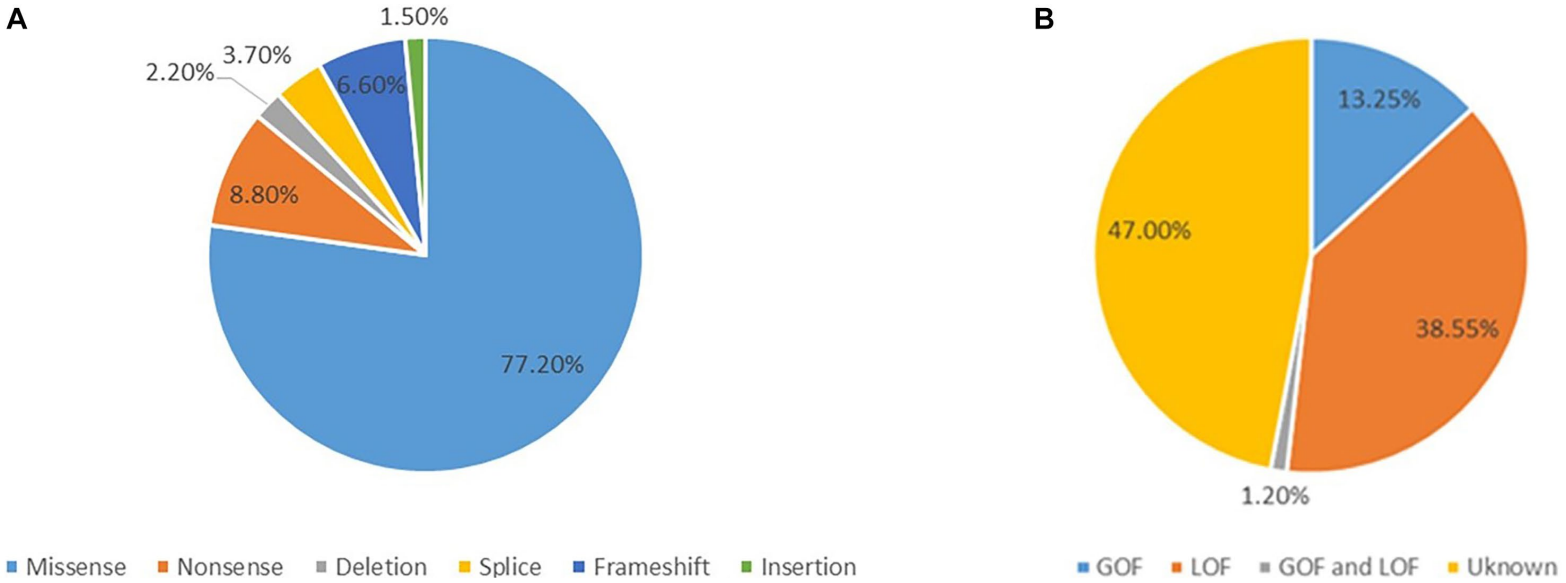
**(A)** The distribution of the variants related to epilepsy. **(B)** The distribution of the 83 epilepsy-related variants according to the electrophysiological changes.

There were 83 variants reported to relate to epilepsy (Imbrici et al., 2004; Kors et al., 2004; Rajakulendran et al., 2010; Zangaladze et al., 2010; Yamazaki et al., 2011; Damaj et al., 2015; Byers et al., 2016; Reinson et al., 2016; Du et al., 2017; Lv et al., 2017; Epperson et al., 2018; Hayashida et al., 2018; Kothur et al., 2018; Lee et al., 2018; Angelini et al., 2019; Jiang et al., 2019; Yamamoto et al., 2019; Gauquelin et al., 2020; Gudenkauf et al., 2020; Mitta et al., 2020; Stendel et al., 2020; Zhang et al., 2020; Ho et al., 2021; Le Roux et al., 2021; Stubberud et al., 2021; Verriello et al., 2021; Alehabib et al., 2022; Bolte et al., 2022; Hommersom et al., 2022; Krygier et al., 2022; Li et al., 2022; Lipman et al., 2022; Niu X. et al., 2022; Niu Y. et al., 2022) as shown in Supplementary Table 2. The majority of the variants were missense (77.20%) followed by nonsense (8.80%) as shown in Figure 1A. For the 83 variants with functional results, 32 (38.55%) were LOF and 11 (13.25%) were GOF (Figure 1B). Of the 83 variants, 21 were recurrent, including p.A713T (*n* = 10), p.V1392M (*n* = 8), p.V1393M (*n* = 7), p.A710T (*n* = 3), p.L226W (*n* = 3), p.R583* (*n* = 3), p.R279C (*n* = 3), p.E101Q (*n* = 3), p.Y62C (*n* = 3), p.R1352Q (*n* = 3), and so on (Supplementary Figure 2).

#### Genotype–phenotype correlations of the *CACNA1A-*related epilepsy

Some recurrent variants showed correlations with status epilepticus, absence seizures, and myoclonic seizures as summarized in Supplementary Figure 3. Status epilepticus showed an association with GOF variants (72.1% Vs. 15.8%, *p* = 0.000). In contrast, LOF variants revealed an association with absence seizures (2.4% Vs. 47.4%, *p* = 0.000) (Supplementary Table 3). We attempted to investigate whether the location of the variants could influence epileptic manifestations. We found that status epilepticus had a correlation with variants located on S4, S5, and S6 (*p* = 0.000) as well as missense mutations (*p* = 0.000). The hotspots for the status epilepticus included p.A710T, p.I711M, p.A712T, p.A713T, p.V1392M, p.V1393M, p.R1348Q, p.R1349Q, and p.Y62C. There was no correlation between the variant location with absence nor myoclonic seizures (Supplementary Table 4).

**Figure 2:**
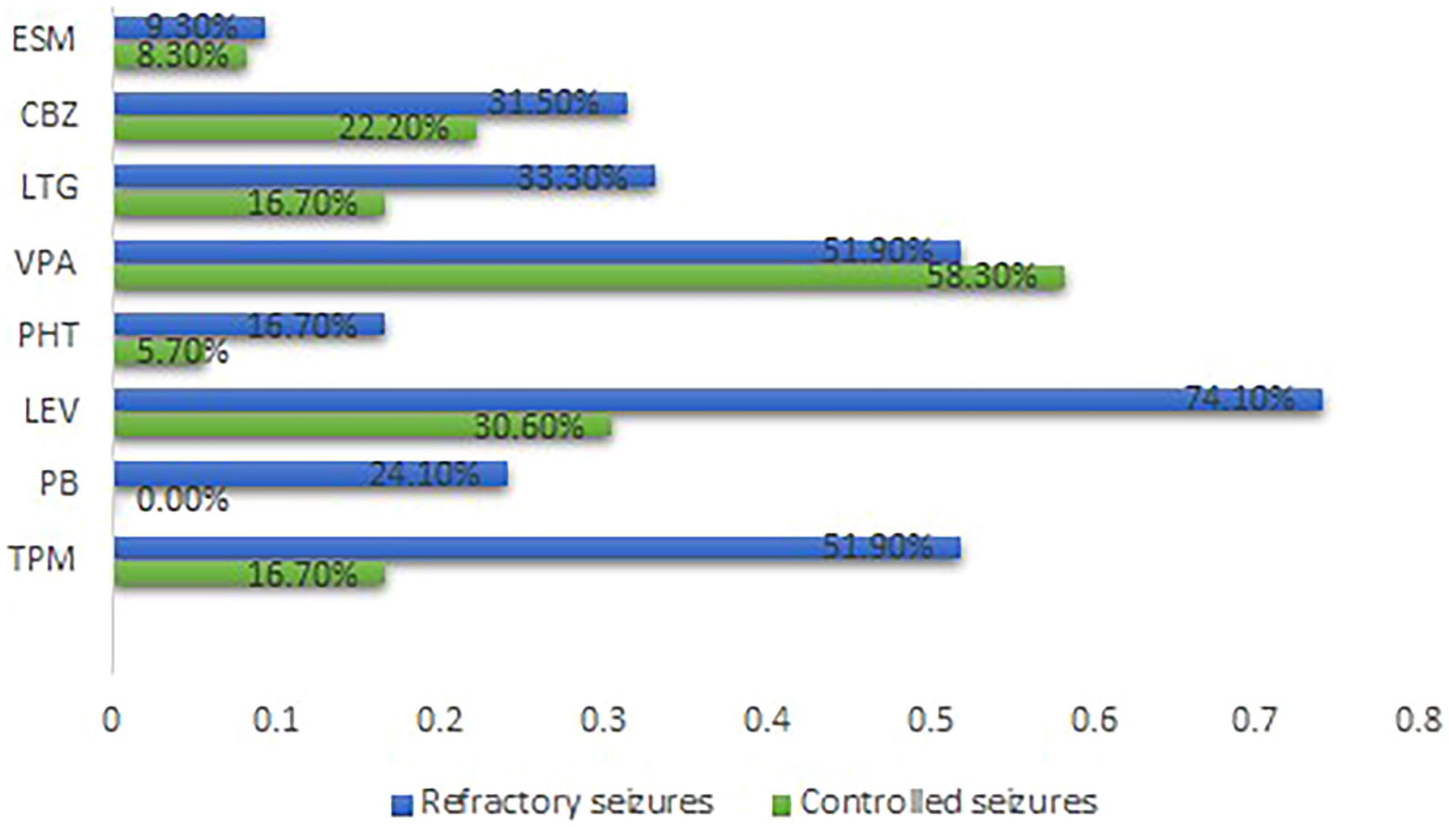
Distribution of the common ASMs used and outcome for all cases Carbamazepine. ESM, Ethosuximide; GOF, Gain-of-function; LEV, Levetiracetam; LTG, Lamotrigine; LOF, Loss-of-function; PB, Phenobarbital; PHT, phenytoin; TPM, Topiramate; VPA, Sodium Valproate.

Epilepsy treatment outcome was not influenced by either electrophysiological changes (GOF or LOF) or type of mutations (missense or nonsense). Nevertheless, patients with refractory epilepsy were more likely to receive topiramate (TPM) (*p* = 0.001), phenobarbital (PB) (*p* = 0.001), and levetiracetam (LEV) (*p* = 0.000) (Supplementary Table 5). Figure 2 summarizes the distribution of the therapies. None of the anti-seizure medications (ASMs) could control seizures among patients in the GOF group. Notably, patients within the refractory seizure group were more likely to receive LEV (*p* = 0.032) (Supplementary Table 6). Supplementary Figure 4 summarizes the distribution of the common ASMs used for the GOF variants and their outcome. None of the ASMs could control seizures among the patients in the LOF group (Supplementary Table 7). Supplementary Figure 5 summarizes the distribution of the common ASMs used for the LOF variants and their outcome.

### *CACNA1A-*related GDD/ID

We found 5 patients from our hospital and 182 individuals from the literature reported to have GDD/ID, and harbor *CACNA1A* pathogenic or likely pathogenic variants. The mean onset age was 25 months, the median was 11 months ±37.1744 SD, the range was 0–144 months. Of the 187 cases, there were 35 (18.72%) from China, 20 (10.70%) from Israel, 19 (10.16%) from France, 17 (9.09%) from France and Belgium, 11 (5.88%) from the USA, 10 (5.35%) from Canada, 10 (5.35%) from Austria, 9 (4.81%) from Spain, 9 (4.81%) from the Netherlands, 8 (4.28%) from Australia, 7 (3.74%) from the UK, 6 (3.21%) from Italy, 6 (3.21%) from Germany, 6 (3.21%) from Japan, 4 (2.14%) from ‘North America’, 2 (1.07%) from Estonia, 1 (0.53%) from Switzerland, 1 (0.53%) from Minnesota, 1 (0.53%) from India, and 5 (2.67%) were from unknown countries (from the Epilepsy Phenome/Genome Project). Of the 99 cases with information about sex, males comprised 41 (41.4%) and females 58 (58.6%) (Supplementary Table 8).

#### Phenotypes of our patients combined with those reported in the literature

**Figure 3:**
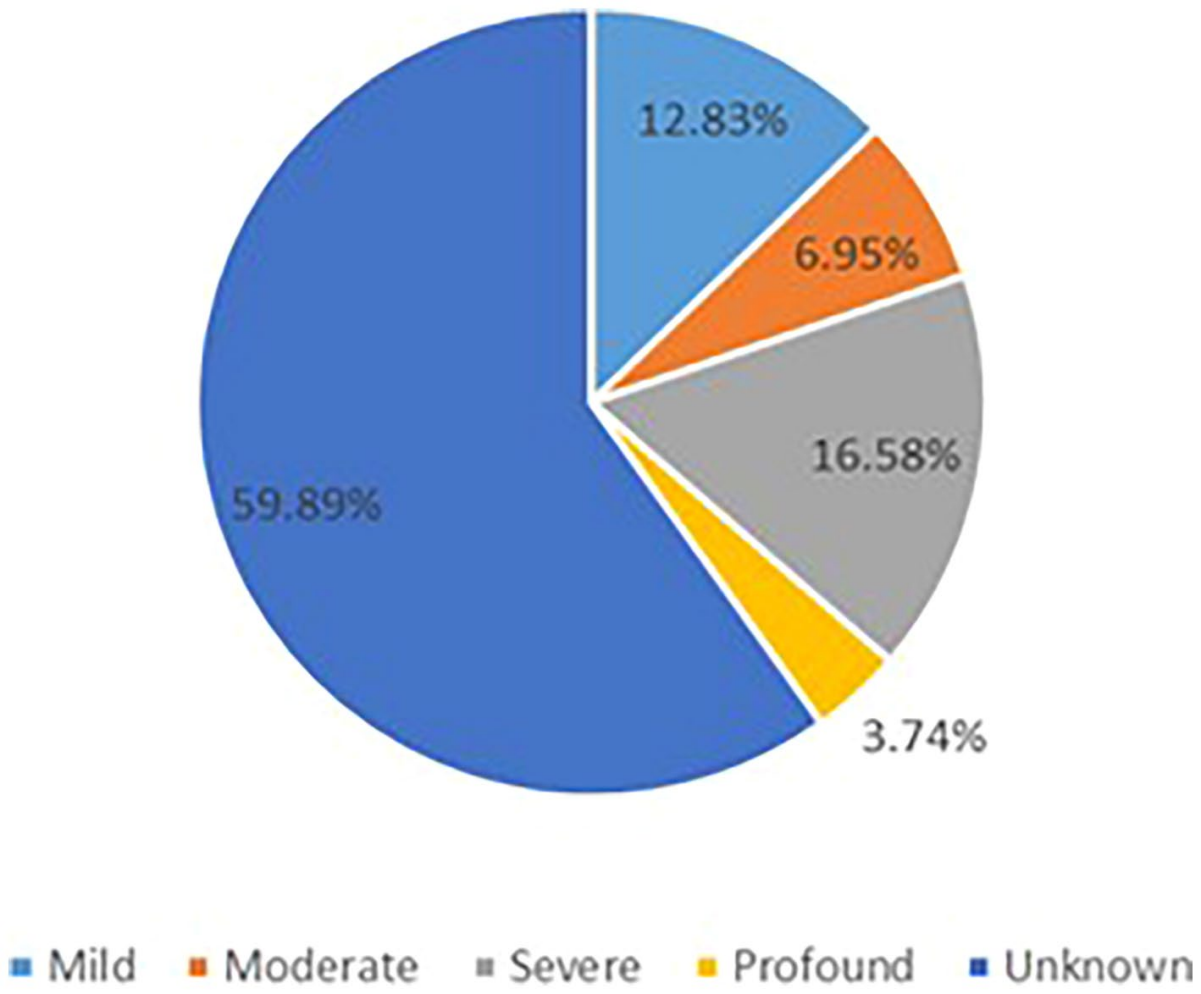
The GDD/ID severity distribution.

The GDD/ID severity distribution is summarized in Figure 3. Progressive cerebellar atrophy (congenital/early onset/isolated) was observed in 46 patients, progressive cerebral atrophy was found in 15 individuals, and hypomyelination/delayed myelination was identified in 4 patients. In addition, progressive optic nerve atrophy was reported in 2 patients, shoulder girdle atrophy in one individual, normal brain imaging findings in 44 cases, and 52 individuals had unknown brain changes (Supplementary Table 8).

#### Genotypes of the *CACNA1A* related GDD/ID

**Figure 4:**
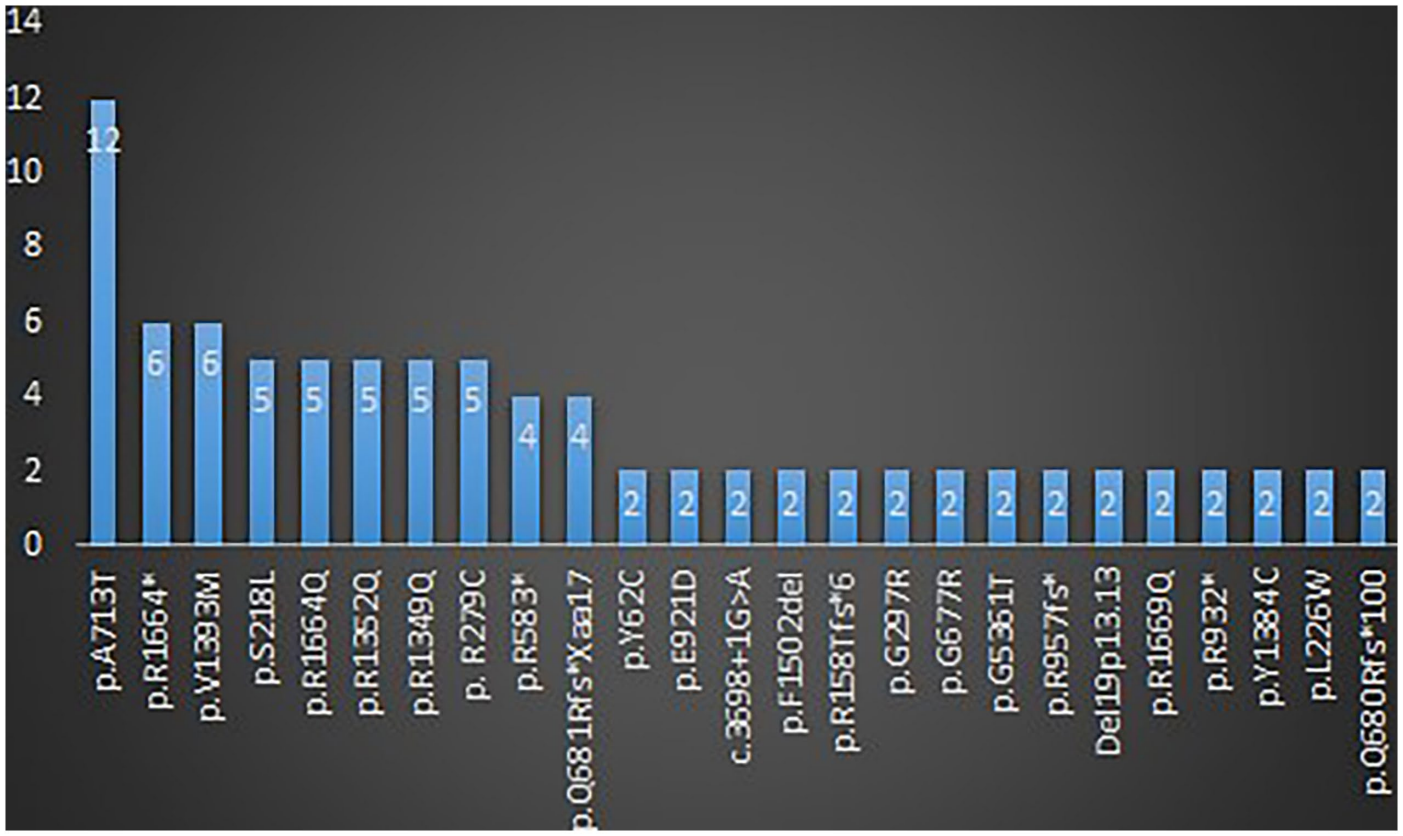
Recurrent variants related to GDD/ID.

**Figure 5:**
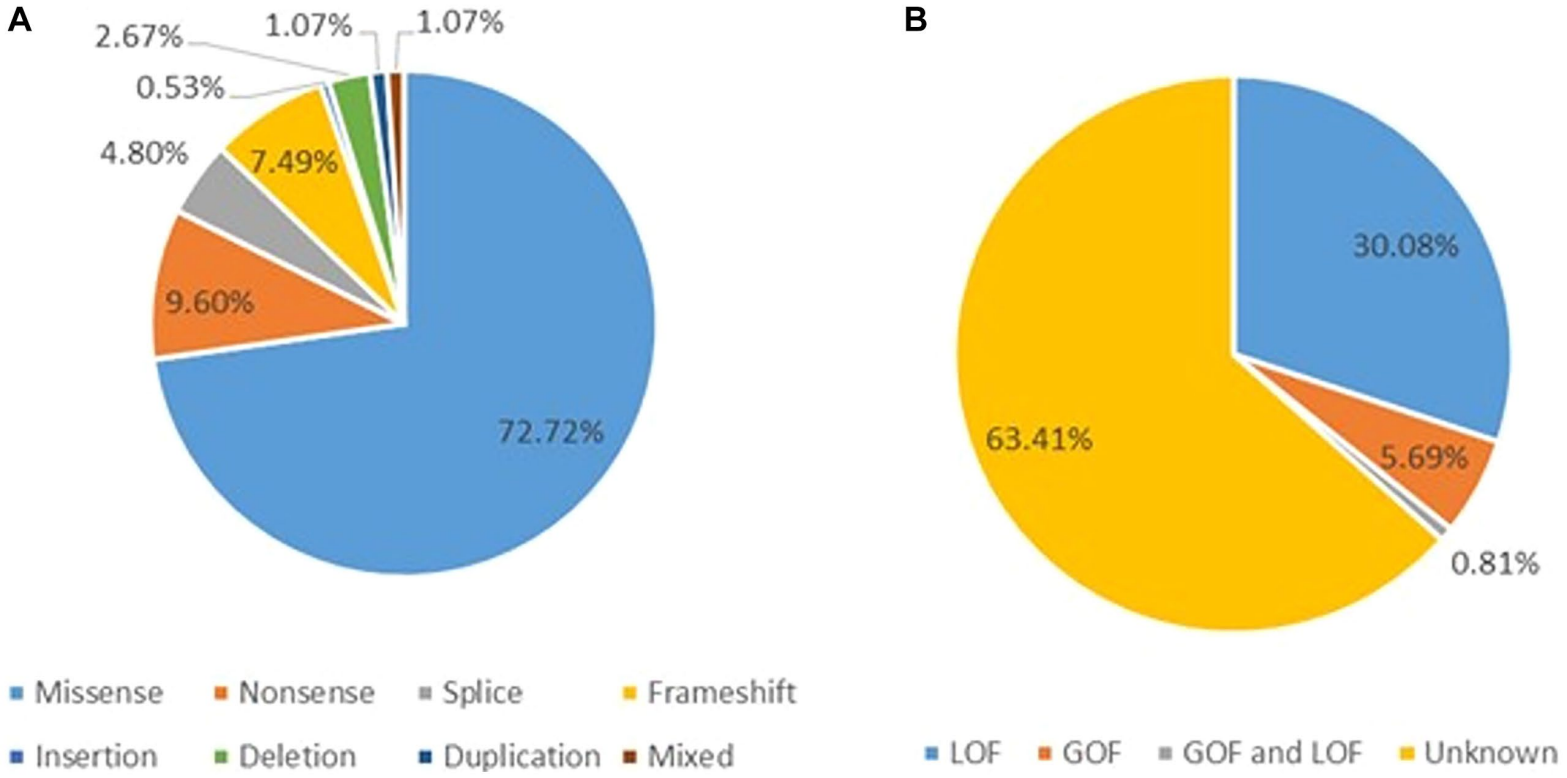
**(A)** Types of the GDD/ID-related mutations. **(B)** The distribution of the GDD/ID-related variants according to the electrophysiological changes.

A total of 123 variants were identified in 187 cases (Fitzsimons and Wolfenden, 1985; Vahedi et al., 2000; Kors et al., 2001; Wada et al., 2002; De Vries et al., 2008; Guerin et al., 2008; Bertholon et al., 2009; Blumkin et al., 2010, 2015; Jung et al., 2010; Mantuano et al., 2010; Rajakulendran et al., 2010; Riant et al., 2010; Carreño et al., 2011; Freilinger et al., 2011; Naik et al., 2011; Yamazaki et al., 2011; Epi4K Consortium et al., 2013; Ohba et al., 2013; Bürk et al., 2014; García Segarra et al., 2014; Bahamonde et al., 2015; Damaj et al., 2015; Epi4K Consortium, 2016; Reinson et al., 2016; Tantsis et al., 2016; Weyhrauch et al., 2016; Balck et al., 2017; Luo et al., 2017; Epperson et al., 2018; Hayashida et al., 2018; Humbertclaude et al., 2018, 2020; Kothur et al., 2018; Angelini et al., 2019; Indelicato et al., 2019; Jiang et al., 2019; Kashimada et al., 2019; Tyagi et al., 2019; Yamamoto et al., 2019; Gauquelin et al., 2020; Gudenkauf et al., 2020; Meloche et al., 2020; Zhang et al., 2020, 2021; Arteche-López et al., 2021; Gandini et al., 2021; Gur-Hartman et al., 2021; Ho et al., 2021; Le Roux et al., 2021; Martínez-Monseny et al., 2021; Saathoff et al., 2021; Stubberud et al., 2021; Alehabib et al., 2022; Bolte et al., 2022; Gajam et al., 2022; Grosso et al., 2022; Hommersom et al., 2022; Li et al., 2022; Mellone et al., 2022; Niu X. et al., 2022; Niu Y. et al., 2022; Wong-Spracklen et al., 2022; Yuan et al., 2022) as shown in Supplementary Table 8. There were 25 recurrent variants which have been summarized in Figure 4. The type of mutations have been summarized in Figure 5A. The distribution of the functional changes of the variants has been summarized in Figure 5B.

#### Genotype–phenotype correlations of the *CACNA1A* related GDD/ID

**Figure 6:**
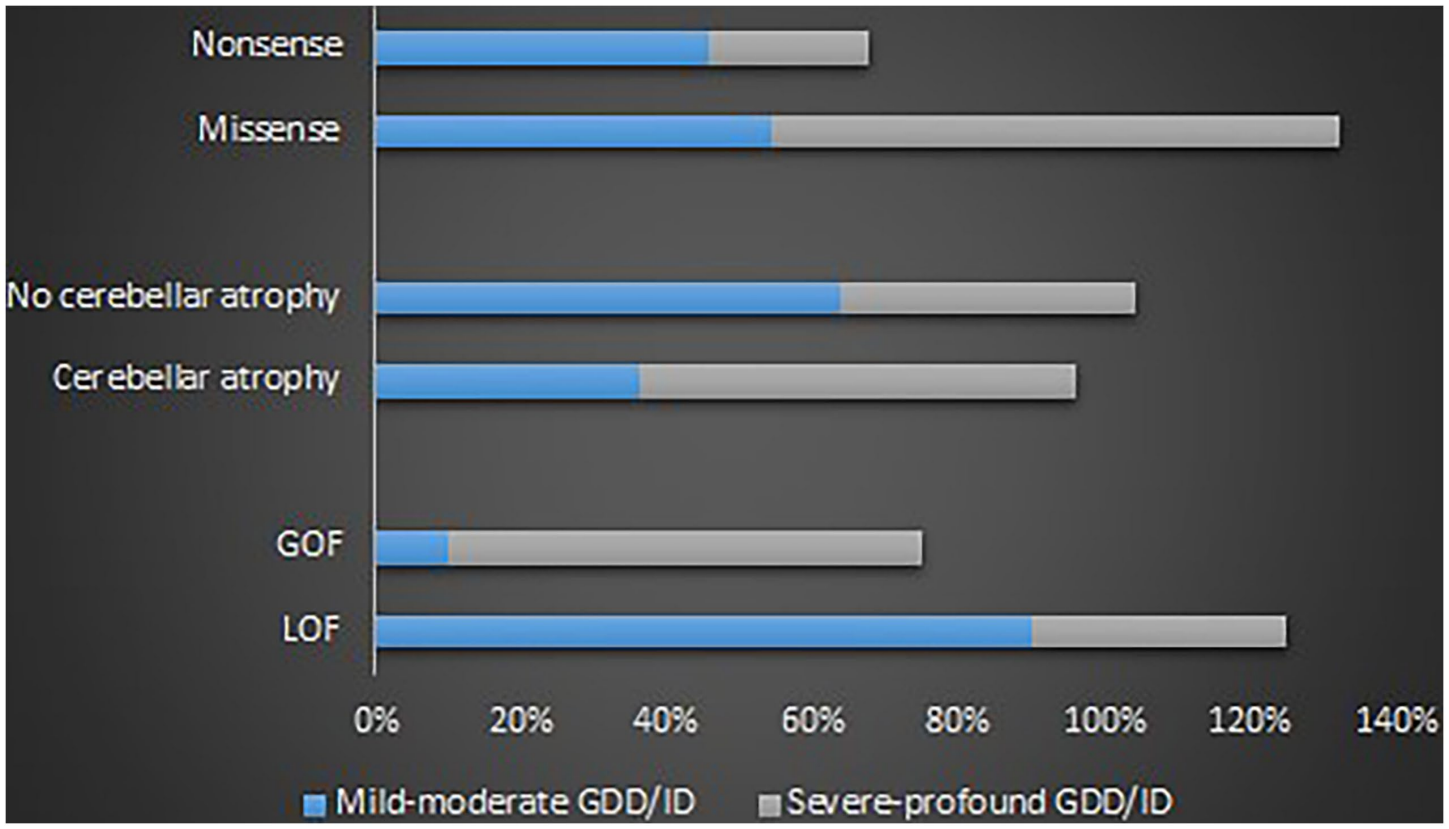
Proportions of the determinants of the GDD/ID severity.

**Figure 7:**
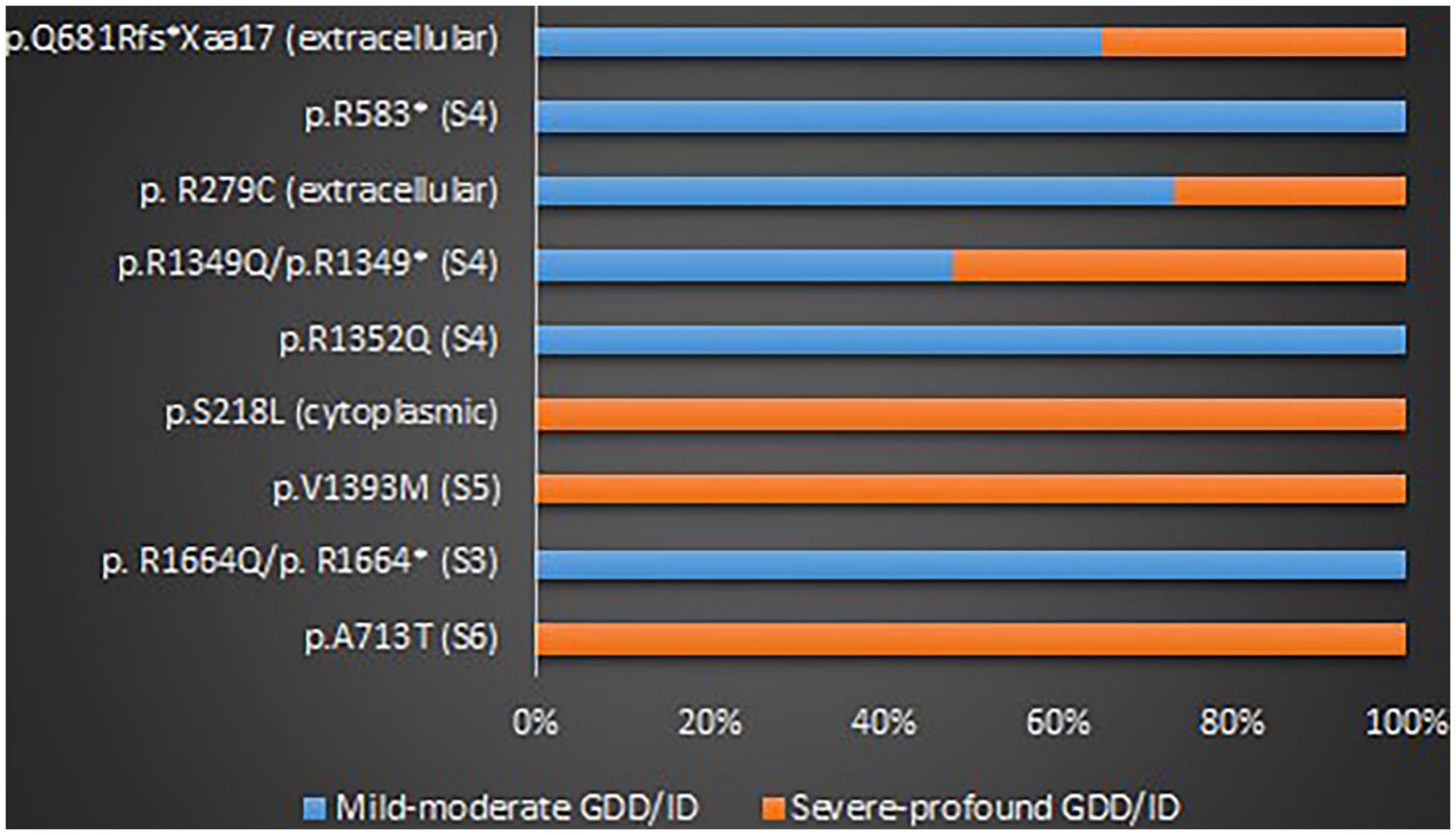
The proportions of the recurrent variants in different GDD/ID severity.

GOF variants showed a substantial relationship with severe – profound GDD/ID, in contrast, LOF variants displayed a correlation with mild–moderate GDD/ID (*p* = 0.001). Cerebellar atrophy was observed more in patients with severe – profound GDD/ID although the result was not statistically significant (*p* = 0.061). Missense mutations were related to severe–profound GDD/ID, however, results were not significant (*p* = 0.072). Supplementary Table 9 and Figure 6 below summarize this information. The p.A713T variant located on S6 with a GOF effect showed a correlation with severe – profound GDD/ID (*p* = 0.003). Supplementary Table 10 and Figure 7 summarize this information.

### *CACNA1A*-related ASD

We found a total of 18 cases that presented with ASD. Ten were males, 5 were females, and 3 had unknown sex. The onset age was unclear for all cases, but mostly during childhood according to their recent ages.

#### General information of our cases plus those reported in the literature

In addition to ASD, 16 had epilepsy, 13 cases had GDD/ID, 9 were hypotonic, 8 had non-episodic ataxia, 3 had ADHD, and 2 had episodic ataxia (Supplementary Table 11).

#### Genotypes of the *CACNA1A* related ASD

**Figure 8:**
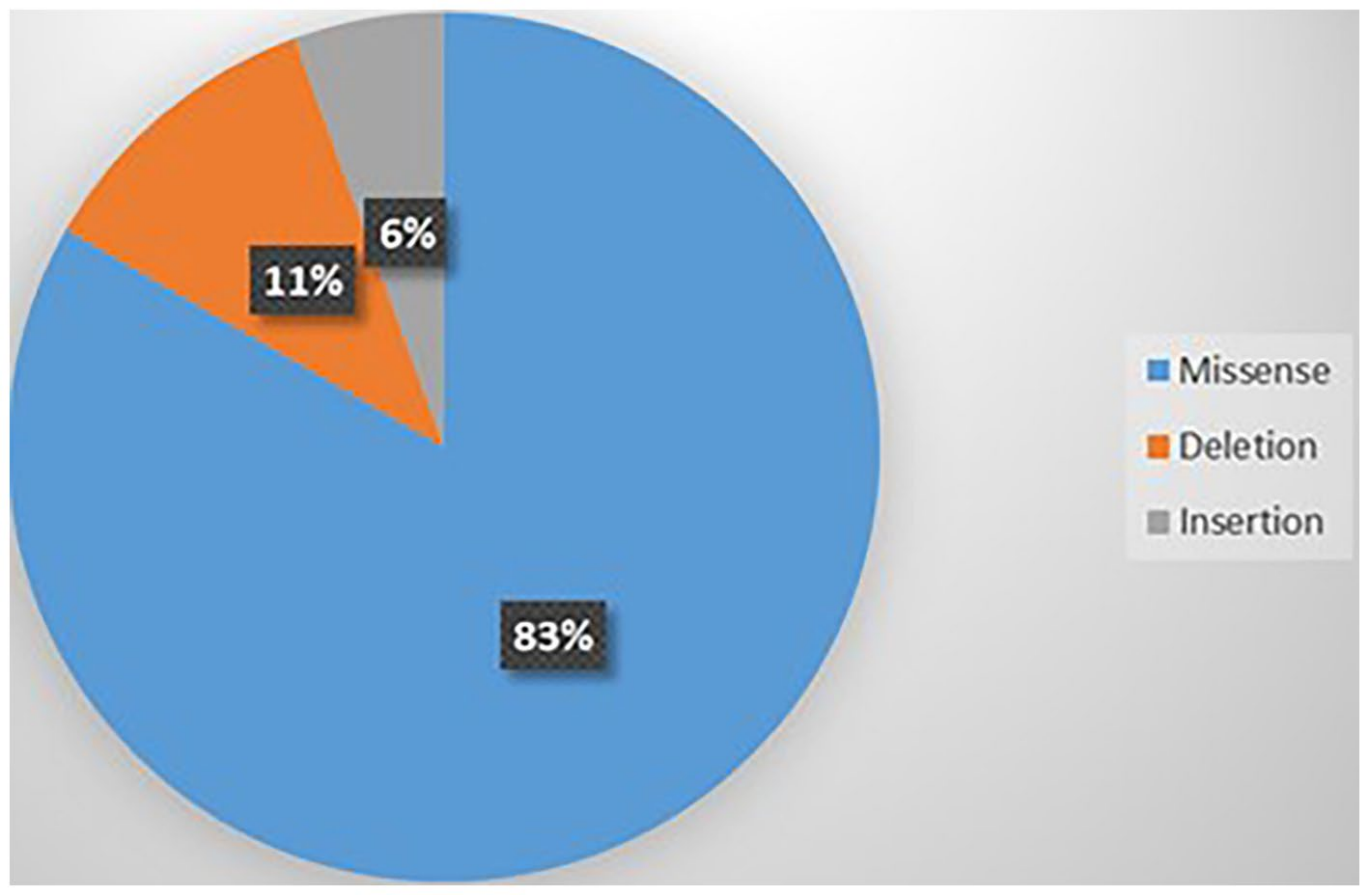
Types of mutations related to ASD.

Thirteen variants were identified in those 18 cases including p.V1392M (*n* = 3), p.A712T (*n* = 2), p.E533K (*n* = 2), p.R1278Ter (*n* = 2), p. G701R (*n* = 1), p.Y62C (*n* = 1), p.R279C (*n* = 1), p.R1348Q (*n* = 1), p.I711M (*n* = 1), p.G700E (*n* = 1), p.S1798L (*n* = 1), p.I1707T (*n* = 1), and p.P2312_ Q2313ins (*n* = 1) (Vila-Pueyo et al., 2014; Damaj et al., 2015; Meloche et al., 2020; Gur-Hartman et al., 2021; Martínez-Monseny et al., 2021). Supplementary Table 11 summarizes this information. The most common type of mutation was missense (83%) as shown in Figure 8. Of the 13 variants, 4 were recurrent; p.V1392M (*n* = 3), p.A712T (*n* = 2), p.E533K (*n* = 2), and p.R1278Ter (*n* = 2) as shown in Supplementary Figure 6. Most of the variants were GOF (53.84%), followed by LOF (30.76%), and 15.38% were unknown (Supplementary Figure 7). Variants were located on S6 (*n* = 6), S5 (*n* = 4), and S2 (*n* = 4).

### *CACNA1A*-related epilepsy, GDD/ID, and ASD

**Figure 9:**
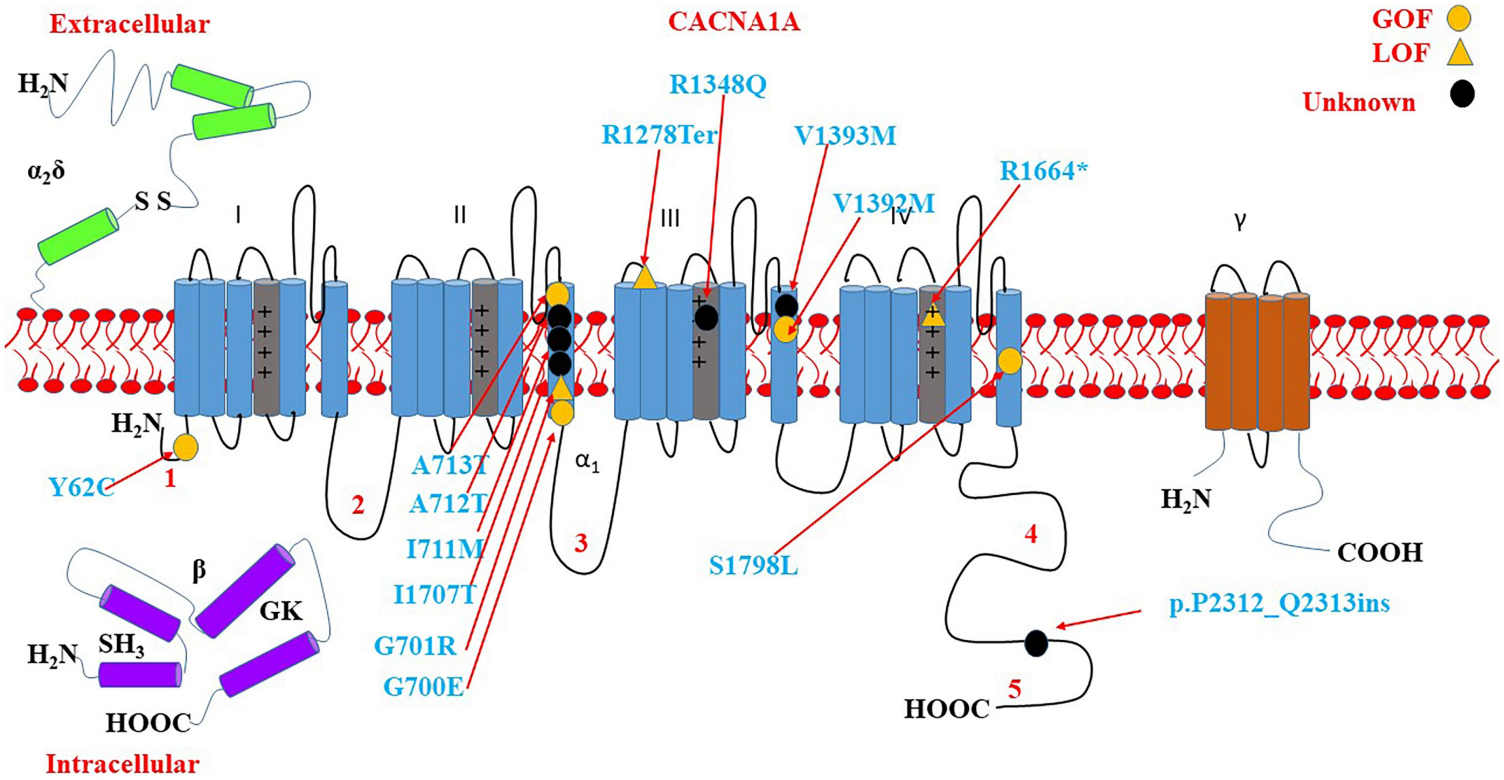
Location of the identified *CACNA1A* amino acid substitutions related to GDD/ID, epilepsy, and ASD. There is a cluster of 5 critical residues in the S6 domain II. Round yellow dots represent gain-of-function variants. Triangular yellow dots represent loss-of-function variants. Round black dots represent unknown functional change variants.

Some of the variants have been reported to associate with epilepsy, GDD/ID, and ASD. The variants include p.A713T, p.R1664*, p.P2312_Q2313ins, p.V1393M, p.Y62C, p.V1392M, p.A712T, p.R1348Q, p.I711M, p.G700E, p.S1798L, p.I1707T, and p.R1278Ter. There is a cluster of six critical residues in the S6 domain II. Most of the variants are located on S4 and S6 as shown in Figure 9.

## Discussion

### *CACNA1A*-related epilepsy and mechanisms

*CACNA1A* is related to a wide range of phenotypes including GDD/ID, epilepsy, and ASD. Different forms of epilepsy including status epilepticus, absence seizures, juvenile myoclonic seizures, and EE have been reported. More than a third of the cases had provoking factors for the seizures/status epilepticus. Status epilepticus correlated with GOF variants whereas, LOF variants were associated with absence seizures. Status epilepticus also showed correlations with missense mutations. We could find some hotspots for the status epilepticus, and variants on S4, S5, and S6 showed a statistically substantial correlation with status epilepticus. More than half of the cases remained with refractory epilepsy. Refractory cases in both GOF and LOF groups were more likely to receive TPM, PB, and LEV. For the GOF patients, individuals within the refractory seizure group were more likely to receive LEV. *CACNA1A* variants caused early deaths in six cases. Most of the reported cases originated from China, the USA, France and/or Belgium, and Korea, suggesting the possibility of the presence of racial disparity. Nevertheless, this finding could also signify that these countries perform more genetic tests compared to other countries in the world.

Diverse forms of epilepsy including status epilepticus, absence seizures, juvenile myoclonic seizures, and EE were related to both GOF, LOF, and mixed GOF and LOF variants. Epilepsy could present a few hours after delivery, approximately half of the patients presented with status epilepticus, and 37.7% had provoking factors. We could observe genotype–phenotype correlations. Status epilepticus and myoclonus showed correlations with GOF variants whereas, LOF variants were linked with absence seizures. The status epilepticus was often focal which could be generalized later. Of interest, patients carrying mutations located on S4, S5, and S6 were more likely to have status epilepticus which corresponds with the specific function of these transmembrane helices. The S4 helix is positively charged, therefore, it is in charge of controlling voltage-dependent activation. The loop between S5 and S6 has negatively charged residues that produce the selectivity filter (Simms and Zamponi, 2014). The hotspots for the status epilepticus include p.A710T, p.I711M, p.A712T, p.A713T, p.V1392M, p.V1393M, p.R1348Q, p.R1349Q, and p.Y62C. It is known that an excessive calcium ions influx through NMDARs can lead to glutamate excitotoxicity as observed in neurodegenerative disorders (Stanika et al., 2012). Moreover, excessive build-up of calcium ions in the neuronal mitochondria can lead to excessive firing like in epileptic seizure, and subsequently resulting in neuronal death (Kramer and Bressan, 2018). A patient diagnosed with intractable epilepsy as well as early stroke, and yet carrying p.L1692Q was found to have mitochondrial depletion (according to muscle biopsy), and an electron transport chain study showed a reduction in several respiratory chain complex activities (Gudenkauf et al., 2020). Noteworthily, *CACNA1A*-related epilepsy has some provoking factors including fever for most of the cases, infection, mild head trauma, stress, excitation, bathing, climatic changes, excitation, agitation, and traveling which somehow present like mitochondrial diseases.

The p.R1667P mutation with mixed GOF and LOF effects has been observed in two children (Grosso et al., 2022). The first patient presented with focal seizures, hypotonia, severe congenital ataxia, dysarthria, and severe cerebral edema which led to death at 5 years of age. The clinical manifestations of the first patient mimicked the autoimmune encephalitis. Post-mortem brain biopsy revealed atrophic cerebellar vermis, loss of Purkinje cells, and hemorrhagic lesions involving the cerebral cortex (Gauquelin et al., 2020). The second patient presented with GDD, microcephaly, pontocerebellar hypoplasia, and progressive cerebellar ataxia (Grosso et al., 2022). The two cases with the same mutations and yet diverse clinical severity or prognosis suggest the complexity of the balance of GOF and LOF changes on the Cav2.1 channel as suggested before (Grosso et al., 2022).

More than half of the cases remained with refractory epilepsy. We have observed that refractory cases for both GOF and LOF were more likely to receive TPM, PB, and LEV. Besides, LEV showed an association with refractory seizures within the GOF group. Whether LEV can worsen seizures is not clear now as many ASMs were prescribed, and it is hard to tell which of them improved or worsened seizures. LEV has been shown to inhibit N-type calcium channels, ryanodine receptor (RyR), and IP3 receptors (IP3R) – activated calcium-induced calcium release (CICR) in hippocampal neurons (Nagarkatti et al., 2008). LEV can also minimize seizures by inhibiting synaptic vesicle glycoprotein 2A (SV2A) (Lynch et al., 2004; Gillard et al., 2006). Notably, LEV has been reported to be effective for generalized tonic–clonic seizures, focal seizures, and generalized myoclonic seizures (Abou-Khalil, 2019). The inability of the LEV to control seizures might be explained by the fact that it has less effect on the Cav2.1 channel. The ability of LEV to worsen seizures might be due to its capacity to interfere with other channels or receptors needed for the regulation of the calcium ions homeostasis. PB works by binding the GABA-A receptor and thus prolongs the opening of the associated chloride channel leading to seizure inhibition. It has been reported that PB is effective against focal seizures and generalized tonic–clonic seizures but is not effective against absence seizures (Abou-Khalil, 2019). Besides, the parenteral solution seems to be effective for status epilepticus (Abou-Khalil, 2019). LTG has been proposed to work on the Cav2.1 channel due to its observed ability to control seizures in very few cases that received several ASMs (Byers et al., 2016; Le Roux et al., 2021). However, we could not find any link between LTG and seizure control in this study. Therefore, the efficacy of LTG on a few *CACNA1A* patients need to be interpreted cautiously because several ASMs were prescribed. The regulation of the calcium ions in the cell is very complex and depends on several channels, transporters, and pumps within organelles and does not depend only on VGCCs.

*Cacna1a* missense mutation mouse model of absence seizures revealed that the cerebellar neurons are powerful regulators of the pathological oscillations in the thalamocortical system (Kros et al., 2015). Although the Cav 2.1 channel deletion lead to epilepsy and ataxia in human, the Cav 2.1 channel deletion in GABAergic interneurons from the medial ganglionic eminence affects cortical inhibition leading to generalized seizures in Nkx2.1Cre; *Cacna1ac/c* mice. Whereas, the Cav 2.1 channel deletion in somatostatin-expressing interneurons does not cause seizures, signifying a vital role of parvalbumin-expressing interneurons (Danzer, 2019). The deletion of the *Cacna1a* in adult mice reproduced the absence epilepsy phenotype by different thalamic bursting mechanisms suggesting that Cav2.1 channels are important for preserving standard thalamocortical oscillations as well as motor regulation in the adult brain (Miao et al., 2020). Tamoxifen prompted adult onset ablation of the *Cacna1a* in layer VI pyramidal neurons of mice that exhibited spontaneous absence seizure phenotype similar to human (Bomben et al., 2016). There is a downregulation of CACNA1A and GABRD proteins expression in the cortical region of the rat brain during epileptogenesis suggesting that the impaired learning and memory of animals might be linked with the dysregulation of both proteins (Kumar and Sharma, 2020). About 90% knockdown of Cacna1a in Larval Zebrafish (absence seizure model) on the protein level induced epileptiform-like discharges in the optic tectum of larval zebrafish brains, and the incubation with ASMs (VPA, ethosuximide, LTG, and TPM) significantly decreased the number and duration of epileptiform-like discharges (Gawel et al., 2020). The Cav2.1 mutation impairs GABAergic inhibition, resulting in abnormal discharges in the hippocampi of epileptic mice without tg (Nakao et al., 2015).

Sudden unexpected death in epilepsy (SUDEP) is a lethal epilepsy complication. A total of six patients died at a young age (from 3 months – 5 years). *CACNA1A* gene compound mutations (p.T1439R, and p.A158Tfs) were identified in two siblings, and they led to the death of one patient at the age of 5 years (Reinson et al., 2016). Both children developed daily recurrent seizures from the age of 4 months which were accompanied by very severe hypotonia, hypokinesia, and GDD. The patients had small corpus callosum, diffuse hypomyelination, as well as progressive cerebral, cerebellar, and optic nerve atrophy. At the age of 5 years, both siblings were blind and bedbound with profound GDD. The elder sister developed significant muscular atrophy and rigidity, and she died at 5 years of age. A nonsense p.R932* was identified in four newborn siblings who died between 3 and 6 months of life (Arteche-López et al., 2021). All four newborns presented with severe hypotonia, encephalopathy, seizures, and mild dysmorphic features. A *de novo* heterozygous pathogenic variant in the *CACNA1A* gene p.R1667P (mixed GOF and LOF variant) was also found in a patient who presented with very severe congenital ataxia and died at the age of 5 years as described in detail in the previous paragraph (Gauquelin et al., 2020). The p.A951V and p.P2421V variants in *CACNA1A* have been reported as risk factors for SUDEP (Coll et al., 2016). Transgenic mice unveiled the main role of the cortical neuronal downregulation and brainstem spreading depolarization in the occurrence of the SUDEP (Loonen et al., 2019). The optogenetic stimulation of the colliculi in *Cacna1a* mice mimicked the SUDEP process (Cain et al., 2022).

### *CACNA1A*-related GDD/ID and mechanisms

The majority of the reported GDD/ID cases originated from China, France and/or Belgium, Israel, the USA, Canada, and Austria. Similar to the geographical distribution of *CACNA1A*-related epilepsy, there could be a racial disparity in the distribution of *CACNA1A*-related GDD/ID. However, it is important to consider the possibility that these phenomena could be due to genetic testing bias. Interestingly, GOF, LOF, and mixed GOF and LOF variants of this gene were associated with mild to severe GDD/ID, and more than half of the cases presented with moderate to profound GDD/ID. We have identified some hotspots for the ID/GDD including p.A713T, p.S218L, p.R1664Q, p.V1393M, p.R279C, p.R1352Q, p.R1349Q, p.V1396M, and p.L226W. GOF variants were associated with severe – profound GDD/ID, in contrast, LOF variants showed an association with mild to moderate GDD/ID. The possible explanation for this finding is, GOF variants might induce neuronal death. Cerebellar atrophy was linked with severe – profound GDD/ID. The p.A713T variant located on S6 with a GOF effect showed a correlation with severe to profound GDD/ID. As we know, S6 forms a pore that allows calcium ions influx through the neurons, therefore, GOF variants might have led to calcium ions overload leading to neuronal overexcitability and death. Therefore, there is a probability that excessive calcium ions influx led to neuronal cell death leading to severe GDD/ID.

The calcium ion signaling process is crucial for synaptic plasticity, learning, and memory. In the Cav2.1 model of schizophrenia (Drosophila), memory impairment was accompanied by a reduction in calcium ions transients at the presynaptic terminals suggesting that loss of the Cav2.1 channel function can lead to cognitive and behavioral deficits (Hidalgo et al., 2021). The CAG repetitions in the *CACNA1A* gene are related to cognitive function in old age plus brain volume changes (Gardiner et al., 2019). Homozygous tottering (tg) mice with a *Cacna1a* mutation with LOF effect exhibited impaired learning in Pavlovian eyeblink conditioning implying that calcium ion homeostasis in neurons is very crucial for learning and memory formation (de Oude et al., 2021). The *Cacna1a* mutant mice tottering (tg) exhibited impaired hippocampus-related memory and synaptic plasticity, similar to the human phenotype (Nakao et al., 2022). Neonatal lesions in the cerebellar system of the *Cacna1a* Rolling mouse Nagoya mice exhibited sensorimotor disturbances similar to children with cerebellar lesions signifying the importance of the cerebellum and its connections in the regulations and development of motor functions (Lalonde and Strazielle, 2015). Moreover, it has been hypothesized that the cerebellum has a role to play in cognition (Shipman and Green, 2019).

Calcium channel blockers (verapamil) showed partial efficacy in the treatment of hemiplegic migraine in individuals with GOF variants, however, there was no comment given regarding its effects on ID (Tantsis et al., 2016). A conditional (forebrain specific) Cav2.1 knock-out mouse exhibited an impairment of synaptic transmission in hippocampal synapses as well as spatial learning and memory deficits (Mallmann et al., 2013). Knock-in mice expressing p.A192G (GOF variant) which is associated with migraine and ID, revealed enhanced hippocampal excitatory transmission and long-term potentiation, however, learning and memory were impaired (Dilekoz et al., 2015). This study demonstrated how unexpected changes in plasticity can affect learning and how heightened neuronal excitability may lead to ID (Dilekoz et al., 2015). A Cav2.1-channel mutant, the heterozygous leaner mouse [tg(la)/+] demonstrated cognitive and motor deficits (Alonso et al., 2008). Ifenprodil (a selective blocker of NMDAR) can block Cav2.1 channels, leading to the reduction of presynaptic excitatory synaptic transmission (Delaney et al., 2012). The blockage of Cav2.1 channels by D1-like dopamine receptors led to low glutamate release into the cholinergic basal forebrain neurons of immature mice (Momiyama and Fukazawa, 2007; Momiyama, 2010).

### *CACNA1A*-related ASD and mechanisms

ASD is also a common phenotype that can be observed in *CACNA1A* mutations. The commonest causative variants were those with the GOF effect, however, how GOF variants lead to ASD is not clear at present. *CACNA1A* rs7249246 and rs12609735 have been linked with ASD in the Chinese Han population (Li et al., 2015). *CACNA1A* single nucleotide polymorphisms are among the top causes of ASD in all VGCCs (Liao and Li, 2020). Homozygous Groggy dams carrying *Cacna1a* missense mutation have no concern for their babies leading to their deaths, which somehow present like ASD (Kawakami et al., 2020). *Auts2* knockout mice have small and malformed cerebella with reduced expression of Cacna1a protein, and impaired motor learning and communication (Yamashiro et al., 2020). Since Cacna1a is a regulator of the synapse development in Purkinje cells, it was suggested that AUTS2 is needed for the typical development of Purkinje cells synapses, therefore, AUTS2 impairment can lead to cerebellar dysfunction linked to ASD (Yamashiro et al., 2020).

### *CACNA1A*-related epilepsy, GDD/ID, and ASD

We have observed an overlap of the genotypes and phenotypes. Notably, most of the variants are located on S4 and S6; the critical regions that regulate the channel gating and calcium ions influx to the cells (Kessi et al., 2021). This suggests that epilepsy, GDD/ID, and ASD might have common underlying mechanisms.

## Conclusion

*CACNA1A* variants are related to a wide range of neurodevelopmental disorder phenotypes including epilepsy, GDD/ID, and ASD. *CACNA1A-*related GDD/ID range from mild to profound, and more than half of the patients present with moderate to profound ID/GDD. The p.A713T variant located on S6 with a GOF effect showed a correlation with severe to profound GDD/ID. More than half of *CACNA1A*-related epilepsy is refractory. The most common epileptic manifestation is status epilepticus which correlates with variants located on S4, S5, and S6. Epilepsy treatment outcome is not influenced by either the electrophysiological changes or mutation types.

### Study limitations

There is no prospective study that assessed the potential treatment for epilepsy, therefore, it is difficult to provide a proper conclusion. Future prospective studies are needed to know more about the natural history of *CACNA1A*-related disorders and potential therapies. The literature review is prone to selection and publication bias. In addition, we might have missed some data due to literature retrieval methodology.

## Data availability statement

The datasets presented in this study can be found in online repositories. The names of the repository/repositories and accession number(s) can be found in the article/Supplementary material.

## Ethics statement

The studies involving human participants were reviewed and approved by the study including all methods adhered to the tenets of the Declaration of Helsinki and received approval from the Institutional Review Board and Research Ethics Committee of Xiangya Hospital, Central South University, Changsha, Hunan. Written informed consent to participate in this study was provided by the participants’ legal guardian/next of kin.

## Author contributions

MK conceptualized, designed the study, performed literature review, collected and analyzed data, and drafted and revised the manuscript. BC and NP aided in data collection and analysis. LF participated in data collection and revised the manuscript. JP, FH, and FY critically reviewed the manuscript for important intellectual content. All authors reviewed the manuscript and approved the submitted version.

## Funding

This work was supported by the Hunan Natural Science Foundation (2021JJQNJJ1515) and the Natural Science Foundation of Changsha City (kq2208384).

## Conflict of interest

The authors declare that the research was conducted in the absence of any commercial or financial relationships that could be construed as a potential conflict of interest.

## Publisher’s note

All claims expressed in this article are solely those of the authors and do not necessarily represent those of their affiliated organizations, or those of the publisher, the editors and the reviewers. Any product that may be evaluated in this article, or claim that may be made by its manufacturer, is not guaranteed or endorsed by the publisher.
