# Supplementary material for: The genotype–phenotype correlations of the CACNA1A-related neurodevelopmental disorders: a small case series and literature reviews
Source: Front Mol Neurosci. 2023 Jul 24;16:1222321. doi: 10.3389/fnmol.2023.1222321 (PMC10406136; doi:10.3389/fnmol.2023.1222321)
Supplement: Supplementary file 1 [file Table_1.docx]

**Supplementary Table 1** Clinical characteristics of six patients with *CACNA1A*-related neurodevelopmental disorders from our hospital

| **Patients** | **P1** | **P2** | **P3** | **P4** | **P5** | **P6** |
| --- | --- | --- | --- | --- | --- | --- |
| Mutation | p. G701R | p. R279C | p. D1644N | p.Y62C | p. L1422Sfs*8 | p. R1664Q |
| Age/Sex | 5y11m/M | 9y8m/F | 13y10m/M | 3y2m/M | 9y10m/F | 8y8m/F |
| Age of seizure onset | - | 5y11m | 10y | 1y20days | - | - |
| Seizure semiology | - | Absence seizures | Focal seizures | Focal seizures | - | - |
| Seizure frequency | - | 1-2 times per day | More than 10 days | Once in 2 weeks | - | - |
| Age of seizure control | - | Ongoing seizures, 1-2 times per day | Seizure attack after 1-2 weeks | - | - | - |
| Presence of the status epilepticus | - | No | No | Yes | - | - |
| **Patients** | **P1** | **P2** | **P3** | **P4** | **P5** | **P6** |
| History of febrile seizures | - | Yes. 3 attacks with temperature >39℃ | No | Yes. Seizures occur mainly in high fever (>38.5℃) | - | - |
| EEG findings | 3-4 Hz slow waves, paroxysm / rhythmic emission in the temporal region | During sleep, 3-4 Hz spike slow waves and multi-spike slow waves. Delta slow waves emission in frontal and occipital regions | Generalized or multifocal spike and slow waves | During sleep, the sharp and slow waves in the frontal, central, and temporal regions were distributed on the left hemisphere. | Bilateral slow waves and slow waves burst on the occipital area | Normal |
| Brain MRI | Cerebellar atrophy | Normal | Cerebellar atrophy, mild enlargement of right ventricle, temporal horn, and occipital cistern. | Abnormal signal in the right hippocampus. | Right choroidal fissure cyst | Progressive cerebellar atrophy |
| **Patients** | **P1** | **P2** | **P3** | **P4** | **P5** | **P6** |
| Treatment strategies | - | Lamotrigine, sodium valproate and acetazolamide | Oxcarbazepine, levetiracetam, and ketogenic diet | Sodium valproate and carbamazepine | - | Acetazolamide |
| Is there a developmental delay before the onset of epilepsy? | Yes | No | Yes | Yes | No | Yes |
| Severity of ID/GDD | Profound | Mild | Profound | Severe | Normal | Mild |
| Motor development | Can neither seat nor walk alone | Poor coordination of independent walking posture (ataxia). | Abnormal gait, dragging on the left side (stroke-like), cannot run, jump and severe motor delay (ataxia) | Has unstable walk (ataxia) | Ataxic gait | Can walk alone but easy to fall and cannot run (ataxia) |
| **Patients** | **P1** | **P2** | **P3** | **P4** | **P5** | **P6** |
| Language development | Can simply express their ideas but inarticulate | Can speak slowly and clear | Can communicate easily, can say sentences, recite poems, but inarticulate | Unconsciously, can call mom and dad | Normal | Repetitive words |
| Family history | No | No | There is a family history of epilepsy | There is a family history of epilepsy | No | No |
| Other clinical features | ASD | Dizziness, loss of consciousness and memory, and ataxia | Fainting attacks and loss of consciousness | ASD. Unstable walking and loss of consciousness | Severe motor delay, and ataxia | Ataxia |
| Physical examination | Slight nystagmus, and hypotonia bilaterally | Nystagmus and ataxia | Left side, grade IV, hypotonia | Undescended left testicle, normal muscle strength, and muscle tone of the limbs | Normal muscle strength and tone of limbs | Nystagmus, ataxic gait, easy to fall, slightly lower muscle tone and reflex. |

**Abbreviations**: ASD: autism spectrum disorder, D: day, EEG: electroencephalograph, F: female, GDD: global developmental delay, ID: intellectual disability, M: male, m: month, MRI: magnetic resonance imaging, P: patient, Y: year.
