# Supplementary material for: The genotype–phenotype correlations of the CACNA1A-related neurodevelopmental disorders: a small case series and literature reviews
Source: Front Mol Neurosci. 2023 Jul 24;16:1222321. doi: 10.3389/fnmol.2023.1222321 (PMC10406136; doi:10.3389/fnmol.2023.1222321)
Supplement: Supplementary file 2 [file Table_2.docx]

**Supplementary Table 2** *CACNA1A* epilepsy-related variants in 130 patients

| **Age of onset/Sex** | **Initial seizure** | **Types of epilepsy** | **Epilepsy trigger** | **Variant** | **Type of mutation** | **Functional effect** | **EEG characteristics** | **All drugs used** | **Seizure reduction drug** | **Seizure free drug** | **Treatment outcome** | **Race/Country of publication** | **References** |
| --- | --- | --- | --- | --- | --- | --- | --- | --- | --- | --- | --- | --- | --- |
| 5y11m/F | Absence seizures | FS | Fever | p. R279C | Missense | LOF | During sleep, 3-4 Hz spike slow waves and multi-spike slow waves. Delta slow waves emission in frontal and occipital regions | LTG, VPA, ACTZ | None | None | Refractory EP | Chinese | Our hospital |
| 10y/M | Focal seizures | - | - | p. D1644N | Missense | GOF | Generalized or multifocal spike and slow waves | OXC, LEV, KD | None | None | Refractory EP | Chinese | Our hospital |
| 1y20d/m | Focal status epilepticus | FS | Fever | p.Y62C | Missense | GOF | During sleep, the sharp and slow waves in the frontal, central, and temporal regions were distributed on the left hemisphere | VPA, CBZ | None | None | Refractory EP | Chinese | Our hospital |
| 7 m/M | Focal status epilepticus | Myoclonic, clonic; GTC | Fever, viral infection | p.V1695L | Missense | UN | Focal and generalized spike-wave discharges | TPM, VPA, LTG LEV, BZD, ACTZ, Pyridoxine,  KD | TPM, VPA, LTG | None | Refractory EP | France and  Belgium | ^[1]^ |
| 17m/M | Focal status epilepticus | Status epilepticus, myoclonic, tonic, atonic | Fever, bath, strong emotion | p.A713T | Missense | GOF | Generalized spike-wave discharges, focal theta rhythmic discharge | TPM, LCS, CBZ,  VPA  LEV, BZD, ACTZ KD | TPM, LCS, CBZ,  VPA | ACTZ | Refractory EP | France and  Belgium | ^[1]^ |
| 24m/F | Focal status epilepticus | Behavioral arrest, autonomic features | Fever, external  climate changes,  stress | p.A713T | Missense | GOF | Focal theta rhythmic discharge | CBZ, TPM, LCS, VPA, LEV, LCS ACTZ, Flunarizine | CBZ, TPM, LCS | None | Refractory EP | France and  Belgium | ^[1]^ |
| 12m/F | Focal behavioral arrest | Automatisms | - | p.D669A | Missense | UN | Focal beta rhythmic discharge | LEV, TPM | LEV, TPM | None | Refractory EP | France and  Belgium | ^[1]^ |
| 12m/M | Absence | Generalized absence | - | p.R1664* | Nonsense | LOF | 3 Hz Generalized spike-wave | TPM, ESM, VPA, LTG | ESM, VPA | TPM | Fluctuation course | France and  Belgium | ^[1]^ |
| 36m/M | G-TC | G-TC |  | p.R583* | Nonsense | LOF | UN | None | None | None | Early seizure freedom | France and  Belgium | ^[1]^ |
| 36m/F | Atypical absence | Atypical absence |  | p.R583* | Nonsense | LOF | Generalized spike-wave (<3 Hz) | TPM, PB, ACTZ | TPM, PB, ACTZ | None | Delayed seizure freedom | France and  Belgium | ^[1]^ |
| 19y/M | Atypical absence | Atypical absence |  | p.R583* | Nonsense | LOF | UN | LEV | LEV | None | Delayed seizure freedom | France and  Belgium | ^[1]^ |
| 2m/F | Focal status epilepticus | Focal clonic |  | p.G939Qfs*128 | Nonsense | LOF | Focal spike-wave discharge | LEV | - | LEV | Early seizure freedom | France and  Belgium | ^[1]^ |
| 38m/M | Atypical absence | Atypical absence |  | p.R279C | Missense | LOF | UN | VPA, OXC CBZ | VPA, OXC | None | Refractory EP | France and  Belgium | ^[1]^ |
| 12m/F | Generalized status epilepticus | - | Fever, bath, menstruation | p.G361E | Missense | UN | Generalized spike-wave (<3 Hz) | OXC, TPM, LTG ESM | OXC, TPM, LTG | None | Refractory EP | France and  Belgium | ^[1]^ |
| 48m/F | Focal status epilepticus | - | Fever | p.A713T | Missense | GOF | Focal beta rhythmic  discharge | LEV, ZNS BZD, VPA, OXC, CBZ Pyridoxine, VNS | LEV, ZNS | None | Refractory EP | France and  Belgium | ^[1]^ |
| 6y/M | Generalized status epilepticus | - | Head trauma, viral infection | p.R1349Q | Missense | GOF | Focal delta periodic  complexes | LEV, Flunarizine | LEV, Flunarizine | None | Fluctuation course | France and  Belgium | ^[1]^ |
| 4.5m/M | Generalized status epilepticus | - | Fever, head trauma | p.S616T | Missense | UN | Focal spike-wave discharge | LEV | LEV | None | Early seizure freedom | France and  Belgium | ^[1]^ |
| 12m/M | Generalized status epilepticus | - | Fever, stress | p.V1393M | Missense | UN | G beta rhythmic  discharge | TPM, VPA, PB CBZ, BZD, LTG, LEV,  ESM | TPM, VPA, PB | None | Refractory EP | France and  Belgium | ^[1]^ |
| 36M/M | Atypical absence | Atypical absence | - | p.A713T | Missense | GOF | Focal theta rhythmic  activity, G beta  rhythmic discharge | LTG, TPM, OXC,  LEV  VPA, CBZ, BZD, ZNS, BZD Pyridoxine, KD,  VNS | LTG, TPM, OXC,  LEV | None | Refractory EP | France and  Belgium | ^[1]^ |
| 36m/F | Absence | Absence | - | p.E1425* | Nonsense | LOF | 3 Hz Generalized spike-wave discharge | TPM, LTG, ESM, VPA, LEV, KD, VNS | TPM, LTG, ESM, KD | None | Refractory EP | France and  Belgium | ^[1]^ |
| 36m/F | Absence | Absence | - | p.W1630* | Nonsense | LOF | 3 Hz Generalized spike-wave | VPA, LTG ESM | VPA, LTG | None | Refractory EP | France and  Belgium | ^[1]^ |
| 5m/M | Focal seizures | Unclassified epileptic encephalopathy | - | p.E101Q | Missense | UN | Left focal epileptiform activity that  spread contralaterally | UN | UN | None | Refractory EP | Italy | ^[2]^ |
| 40y/M | Focal seizures | Unclassified | - | p.M1490_S1491del | Deletion | LOF | Normal | LEV | None | LEV | Seizure free | Italy | ^[2]^ |
| 6y/F | Absence | Genetic generalized epilepsy | - | p.M1490_S1491del | Deletion | LOF | Normal | VPA, ESM | None | VPA, ESM | Seizure free | Italy | ^[2]^ |
| UN/M | Status epilepticus | - | - | p.V1392M | Missense | GOF | UN | UN | UN | UN | Refractory EP | USA | ^[3]^ |
| UN/F | Status epilepticus | - | - | p.V1392M | Missense | GOF | UN | UN | UN | UN | Refractory EP | USA | ^[3]^ |
| UN/F | Status epilepticus | - | - | p.V1392M | Missense | GOF | UN | UN | UN | UN | Controlled | USA | ^[3]^ |
| UN/F | Status epilepticus | - | - | p.V1392M | Missense | GOF | UN | UN | UN | UN | Controlled | USA | ^[3]^ |
| UN/F | Status epilepticus | - | - | p.V1392M | Missense | GOF | UN | UN | UN | UN | Refractory EP | USA | ^[3]^ |
| UN/F | Status epilepticus | - | - | p.V1392M | Missense | GOF | UN | UN | UN | UN | Refractory EP | USA | ^[3]^ |
| UN/F | Status epilepticus | - | - | p.V1392M | Missense | GOF | UN | UN | UN | UN | Controlled | USA | ^[3]^ |
| UN/F | Status epilepticus | - | - | p.V1392M | Missense | GOF | UN | UN | UN | UN | Refractory EP | USA | ^[3]^ |
| UN/M | Status epilepticus | - | - | p.A1507T | Missense | GOF | UN | UN | UN | UN | Refractory EP | USA | ^[3]^ |
| UN/F | Status epilepticus | - | - | p.L1344P | Missense | LOF | UN | UN | UN | UN | Refractory EP | USA | ^[3]^ |
| UN/F | Status epilepticus | - | - | p.D1633N | Missense | LOF | UN | UN | UN | None | Controlled | USA | ^[3]^ |
| UN/F | Status epilepticus | - | - | p.L617S | Missense | GOF | UN | UN | UN | UN | Controlled | USA | ^[3]^ |
| UN/F | Status epilepticus | - | - | p.A712T | Missense | GOF | UN | UN | UN | UN | Refractory EP | USA | ^[3]^ |
| UN/F | Status epilepticus | - | - | p.R1348Q | Missense | GOF | UN | UN | UN | UN | Refractory EP | USA | ^[3]^ |
| UN/M | Status epilepticus | - | - | p.A712T | Missense | GOF | UN | UN | UN | UN | Refractory EP | USA | ^[3]^ |
| UN/M | Status epilepticus | - | - | p.R1348Q | Missense | GOF | UN | UN | UN | UN | Refractory EP | USA | ^[3]^ |
| UN/M | Status epilepticus | - | - | p.I711M | Missense | GOF | UN | UN | UN | UN | Refractory EP | USA | ^[3]^ |
| UN/M | Status epilepticus | - | - | p.G700E | Missense | GOF | UN | UN | UN | UN | Controlled | USA | ^[3]^ |
| UN/M | Status epilepticus | - | - | p.S1798L | Missense | GOF | UN | UN | UN | UN | Controlled | USA | ^[3]^ |
| UN/F | Status epilepticus | - | - | p.R1351Q | Missense | GOF | UN | UN | UN | UN | Controlled | USA | ^[3]^ |
| 21y/F | Juvenile myoclonic epilepsy | GTCS, Myoclonus | - | p.L226W | Missense | UN | Normal | CBZ | None | CBZ | Controlled | Iran | ^[4]^ |
| 16y/F | Juvenile myoclonic epilepsy | GTCS, Myoclonus | - | p.L226W | Missense | UN | Normal | CBZ | None | CBZ | Controlled | Iran | ^[4]^ |
| 16y/M | Juvenile myoclonic epilepsy | GTCS, Myoclonus | - | p.L226W | Missense | UN | Normal | VPA, Ritalin,  Risperid | None | VPA, Ritalin,  Risperid | Controlled | Iran | ^[4]^ |
| 11m | Absence | GTCS, absences | - | p.R1278X | Nonsense | LOF | Generalized theta bursts | VPA, TPM, LEV | None | VPA, TPM, LEV | Controlled | Canada | ^[5]^ |
| 18m | Absence | - | - | p.R957fs | Frameshift | LOF | UN | VPA, LEV, ESM | VPA, LEV | UN | Controlled | Canada | ^[5]^ |
| After birth/F | Status epilepticus | Myoclonic, focal, and secondary generalized | Fever, head trauma | p.A713T | Missense | GOF | Generalized slowing and excessive beta activity and focal discharges | MDZ, LZM | None | IV phenobarbital | Refractory seizures | USA | ^[6]^ |
| 17m/F | Status epilepticus | Focal seizures | Fever | p.C1369R | Missense | UN | Generalized delta waves with multifocal spikes, as well as excessive beta activity. | MDZ, LZM, FosPHT, LEV | None | None | Refractory EP | USA | ^[6]^ |
| UN | Unclassified EP | - | - | p.A952Sfs*115 | Nonsense | LOF | Normal | UN | None | None | UN | Netherlands | ^[7]^ |
| UN | Unclassified EP | - | - | p.T179A | Missense | UN | Normal | UN | None | None | UN | Netherlands | ^[7]^ |
| 3y/F | Focal epilepsy | Simple focal seizures | - | p.G989Rfs*78 | Nonsense | LOF | Diffuse spike-wave,  irregular sharp  and spike  waves | UN | None | VPA | Seizure free | Chinese | ^[8]^ |
| 1y/F | Focal epilepsy | - | - | c.3089 C 1G > A | Splice | LOF | Bilateral  occipital slow spike-waves | UN | None | VPA | Seizure free | Chinese | ^[8]^ |
| 2y/F | Absence seizures | - | Fever | c.4755 C 1G > T | Splice | LOF | Ictal: 10 Absence;  interictal:  paroxysmal 3  HZ slow spike-waves | UN | None | VPA | Seizure free | Chinese | ^[8]^ |
| 6y/M | Absence seizures | - | - | c.6340-1G > A | Splice | LOF | Paroxysmal  generalized  3 Hz slow spike-waves | UN | None | VPA | Seizure free | Chinese | ^[8]^ |
| 11y/F | Focal seizures | Secondary GTC | - | p.R68L | Missense | UN | Right frontal  and temporal  spikes and  focal spike-waves | UN | None | VPA, LTG | Seizure free | Chinese | ^[8]^ |
| 3m/F | Focal seizures | Secondary GTC | - | p.G1322E | Missense | UN | Left parietal  and temporal  sharp waves  and focal spike-waves | UN | None | VPA, LTG | Seizure free | Chinese | ^[8]^ |
| 10y/M | Focal epilepsy | Simple focal seizures | - | p.R1678C | Missense | UN | Bilateral  occipital sharp  waves | UN | None | OXC | Seizure free | Chinese | ^[8]^ |
| 1.5y/M | Focal seizures | Secondary GTC | - | p.S1798L | Missense | GOF | Bilateral  occipital spikes  and focal spike-waves | UN | None | VPA | Seizure free | Chinese | ^[8]^ |
| 4y/F | Focal seizures | GTCS | Fever | p.S1078L  p.E2021K | Missense | UN | Bilateral frontal  and central  sharp waves | UN | None | VPA, OXC | Seizure free | Chinese | ^[8]^ |
| 1y/M | Focal seizures | Complex partial seizure | Fever | p.I1631V  p.P1993L | Missense | UN | Left parietal  and temporal  spikes | UN | None | LEV | Seizure free | Chinese | ^[8]^ |
| UN | Juvenile absence epilepsy | Atypical absence seizure | - | p.Q680RfsTer100 | Splice | LOF | Generalized/occipital discharges | VPA and/or LEV | None | VPA and/or LEV | Controlled | Chinese | ^[9]^ |
| 8 weeks/F | Status epilepticus | Atypical Febrile seizures | Fever | p.L1692Q | Missense | UN | Diffuse background slowing with periods of generalized suppression, focal voltage attenuation in the left hemispheric leads, and multifocal epileptiform discharges. | LEV, VNS | VNS | None | Refractory | USA | ^[10]^ |
| 2d/F | Status epilepticus, EIEE | FS, myoclonic, GTCS | Fever | p.A713T | Missense | GOF | Midline spike-wave, generalized spike-wave, slow spike-waves, polyspike-waves | VPA, LEV | None | None | Refractory | Chinese | ^[11]^ |
| 1y 4m/F | Status epilepticus, DEE | FS, focal motor status epilepticus | Fever | p.V1393M | Missense | UN | Left temporal rapid wave, sharp theta wave, sharp wave | TPM, OXC, LEV | None | OXC, LEV | Refractory | Chinese | ^[11]^ |
| 8y 6mo/F | Absence seizures | - | - | p.Q680ArgfsTer100 | Splice | LOF | 3Hz generalized spike-waves, polyspike-waves | LTG, VPA, CZP, CBZ | None | LTG, | Seizure free | Chinese | ^[11]^ |
| 3m/F | Status epilepticus, DEE | Focal seizures | - | p.G1323E | Missense | UN | Left temporal slowing, left multifocal sharp waves | VPA, TPM | None | VPA, TPM | Seizure free | Chinese | ^[11]^ |
| 1d/F | Status epilepticus, EIEE | GTCS, FS， myoclonic seizures | Fever | p.A711T | Missense | UN | Diffuse slowing， generalized spike-wave, slow spike-waves, polyspike-waves | PB, CZP, VPA, TPM, LEV | None | None | Refractory | Chinese | ^[11]^ |
| 1y/F | Status epilepticus | FS, focal motor | Fever | p.Y62C | Missense | GOF | Left frontal slowing, midline spike-wave | TPM, LEV, VPA | None | LEV | Refractory | Chinese | ^[11]^ |
| 7.5m/F | Status epilepticus, DEE | FS, GTCS | Fever | p.V1393M | Missense | UN | Midline sharp waves, slow spike-waves | CBZ, CZP, VPA, TPM, LEV, ZNS  LEV | None | CZP, VPA, ZNS | Seizure free | Chinese | ^[11]^ |
| 1y6m/M | DEE | FS | Fever | p.I759T | Missense | UN | Multifocal spike-wave, slow spike-waves | LEV | None | None | Refractory | Chinese | ^[11]^ |
| 1m/F | Status epilepticus, EIEE | FS, focal motor status epilepticus | Fever | p.S1469L | Missense | UN | Normal | TPM | None | None | Refractory | Chinese | ^[11]^ |
| 5m/M | Status epilepticus, EIEE | FS, focal motor status epilepticus | Fever | p.V1393M | Missense | UN | Normal | LEV, VPA | None | None | Refractory | Chinese | ^[11]^ |
| 4m/F | Status epilepticus, EIEE | FS, focal motor status epilepticus | Fever | p.R55S | Missense | UN | Diffuse slowing, right hemispheric spike-wave, polyspike-waves | LTG, CLB, VPA, LEV, TPM, CZP, KD | None | CLB, CZP | Refractory | Chinese | ^[11]^ |
| 5m/F | Status epilepticus, EIEE | FS, febrile convulsions, focal motor status epilepticus | Fever | p.Q685X | Nonsense | LOF | Left hemispheric spike-wave, slow spike-waves, poly spike-waves | LEV | None | None | Refractory | Chinese | ^[11]^ |
| 5m/M | EIEE | FS  epileptic spasms | Fever | c.6530-1G＞C | Splice | LOF | Left hemispheric slow wave, sharp wave | TPM, LEV, ZNS | None | None | Refractory | Chinese | ^[11]^ |
| 1y5y/M | Status epilepticus, DEE | FS, focal motor status epilepticus | Fever | p.V1393M | Missense | UN | Left hemispheric slow wave, sharp wave | LEV, OXC, VPA | None | None | Refractory | Chinese | ^[11]^ |
| 3y7m/M | Absence seizures | Absence seizures | - | p.G297R | Missense | UN | 3Hz generalized spike-waves | LTG, VPA, LEV | None | None | Refractory | Chinese | ^[11]^ |
| 8.5m/F | Status epilepticus, DEE | FS, focal motor status epilepticus | Fever | p.V1393M | Missense | UN | Normal | PB, VPA, LEV, OXC, CZP, LTG | PB | None | Refractory | Chinese | ^[11]^ |
| 7y/M | Atypical absence seizures | FS, epileptic spasms, atypical absence seizures | Fever | p.W169X | Nonsense | LOF | Generalized and multifocal spike-wave, slow spike-waves, polyspike-waves, sharp wave | VPA, OXC, LEV, TPM, CZP, perampanel | None | Perampanel | Refractory | Chinese | ^[11]^ |
| 3y11m/M | FS | FS | Fever | p.N283S | Missense | UN | Right frontal and temporal SSW, sharp wave | VPA, LEV, LTG | None | LTG | Refractory | Chinese | ^[11]^ |
| 4y/M | Status epilepticus, DEE | Secondarily generalized tonic–clonic seizures and complex  partial seizures | Head trauma | p. S218L | Missense | GOF | Diffusely slow background and continuous  right temporal rhythmic delta activity. Subsequent EEGs showed  status epilepticus with recurrent right midtemporal seizures. | VPA, intravenous FosPHT | None | Intravenous FosPHT | Seizure free | USA | ^[12]^ |
| 9m/M | Bathing seizures and loss of consciousness | Bathing seizures | Bathing | p.I108V | Missense | UN | Focal discharges  of spikes and slow waves over the right temporal area. | CBZ, VPA | None | CBZ, VPA | Seizure free | Poland | ^[13]^ |
| 2d/M | LGS | Focal, GTC, myoclonic | - | p.A713T | Missense | GOF | Generalized spike-waves | PB, PHT, NTZ, VGB, RUF, VPA, TPM, LVT, CLB, CBZ, | None | None | Refractory | North America | ^[14]^ |
| 6m/M | Status epilepticus, EE | Focal | - | p.G230V | Missense | LOF | Generalized or bilateral focal spike-waves | VPA, FosPHT | None | None | Refractory | North America | ^[14]^ |
| 6m/F | FS,EE | FS, myoclonic | Febrile | p.I1357S | Missense | LOF | Generalized spike-waves, diffuse slowing | PB, VNS, CLB, FELB, LVT, CBD, CBZ, RUF, TPM, VPA, LZP, KD | None | None | Refractory | North America | ^[14]^ |
| 5m/F | EE | Focal, GTC | - | p.V1396M, p.G2314S | Missense | GOF | Right occipital slowing | PB, LVT, PHT, P5P | None | None | Refractory | North America | ^[14]^ |
| 12y/F | TLE | FS | Fever | p.G540R | Missense | LOF | Generalized spike-wave discharge; focal slowing, left temporal emphasis | UN | UN | UN | UN | UK | ^[15]^ |
| 6y/M | Absence seizures | - | - | p. G1105S | Missense | LOF | 3 Hz SWD | UN | UN | UN | UN | UK | ^[15]^ |
| 12m/M | EOEE | Myoclonic epilepsy | - | p.V1808L | Missense | UN | Multifocal spikes and slow waves | MDZ, FosPHT, CLB,  PB, ZNS, LEV, VPA, KD | PB, ZNS, LEV, VPA, KD | UN | Refractory | Japan | ^[16]^ |
| 5y/F | Status epilepticus | Complex partial seizures | Infection | p.S218L | Missense | GOF | Normal | MDZ, CLB | - | MDZ, CBZ, CLB | Controlled | Japan | ^[17]^ |
| 3y/M | Status epilepticus | GTC | Minor head trauma, infection | p.R1352Q | Missense | UN | Sharp focus on the left temporal region. | UN | UN | UN | UN | UK | ^[18]^ |
| 3y/M | EE | Tonic spasms |  | p.W1439R and p.A158Tfs*6 | Nonsense | LOF | Multifocal sharp waves and spike-wave complexes | UN | UN | UN | Refractory | Estonia | ^[19]^ |
| 5y/F | EE | - | - | p.W1439R and p.A158Tfs*6 | Nonsense | LOF | Generalized  spike and slow wave complexes discharges | UN | UN | UN | Died | Estonia | ^[19]^ |
| 4y | Absence | - | - | p.E147K | Missense | LOF | 3 Hz spike-wave discharges | UN | CLP and PHT | UN | Refractory | UK | ^[20]^ |
| 13y | Absence | - | - | p.E147K | Missense | LOF | 3 Hz spike-wave discharges | UN | ACTZ, CBZ | UN | Controlled | UK | ^[20]^ |
| 3.5y/M | Absence | Myoclonic and absence seizures | - | p.R279C | Missense | LOF | Diffuse slow  waves when awake and few rolandic epileptic spikes during sleep | OXC | OXC | UN | Refractory | France | ^[21]^ |
| 5y/M | Status epilepticus | Complex partial seizures. | - | p.I1710T | Missense | UN | Normal | CBZ, PHT | CBZ, PHT | UN | Seizure free | Netherlands | ^[22]^ |
| 1.5y | Complex partial seizure | GTC | Infection, fever | p.I1710T | Missense | UN | Normal | PHT | PHT | UN | Seizure free | Netherlands | ^[22]^ |
| UN | FS | FS | Fever | p.R1352Q | Missense | UN | Not clear | UN | UN | UN | UN | Chinese | ^[23]^ |
| UN | Status epilepticus, FS | FS | Fever | p.R1352Q | Missense | UN | Not clear | UN | UN | UN | UN | Chinese | ^[23]^ |
| UN | Status epilepticus, FS | FS | Fever | p.G701V | Missense | UN | Not clear | LEV, TPM | UN | TPM | Seizure free | Chinese | ^[23]^ |
| UN | Status epilepticus, FS | FS | Fever | p.A713T | Missense | GOF | Not clear | LEV, OXC, TPM | LEV, OXC | TPM | Seizure free | Chinese | ^[23]^ |
| UN | Status epilepticus, FS | FS, focal seizures | Fever | p.V1393M | Missense | UN | Not clear | LEV, TPM | LEV, TPM | UN | Controlled | Chinese | ^[23]^ |
| UN | Status epilepticus, FS | FS, focal seizures, hemiconvulsion-hemiplegia-epilepsy | Fever | p.Y62C | Missense | GOF | Not clear | VPA | VPA | UN | Controlled | Chinese | ^[23]^ |
| UN | Atypical absence seizures | GTCS, FC | - | p.F1814L | Missense | UN | Not clear | LTG, VPA | VPA | LTG, VPA | Seizure free | Chinese | ^[23]^ |
| 50y/UN | Progressive myoclonic epilepsy | - | - | c.6975_6976insCAG, | Insertion | UN | Generalized polyspike-waves or generalized spike-slow complex wave | UN | None | UN | Refractory | Chinese | ^[24]^ |
| 50y/UN | Progressive myoclonic epilepsy | - | - | c.6975_6976insCAG, | Insertion | UN | Generalized polyspike-waves or generalized spike-slow complex wave | UN | None | UN | Refractory | Chinese | ^[24]^ |
| 3y/M | Status epilepticus, absence seizures | - | - | c.1913 + 2T > G | Splice | LOF | Epileptiform discharges | ESM, VPA | ESM, VPA | UN | Controlled | Germany | ^[25]^ |
| 4weeks/M | EIMFS | Tonic, focal | - | p.E101Q | Missense | UN | Multifocal; diffuse  slowing; Right temporoparietal  spikes | LTG, TPM, LEV, PB, MDZ, CLZ, CZP, PHT,  lignocaine, pyridoxine,  propranolol,  pyridoxal-phosphate | LTG, TPM, LEV, PB | None | Refractory | Not clear (Epilepsy Phenome/Genome Project) | ^[26]^ |
| At birth/M | EOEE | Myoclonic | - | p.A713T | Missense | GOF | Multifocal; beta activity;  R > L sharp and slow waves | LTG, VPA, TPM, LEV, PB, CBZ, CZP, MDZ, PHT, DZP | LTG, VPA, TPM, LEV | None | Refractory | Not clear (Epilepsy Phenome/Genome Project) | ^[26]^ |
| 1-2 hours after birth/F | EOEE | Tonic with focal features | - | p.A713T | Missense | GOF | Generalized spike-waves, polyspike-waves, mild  slowing bi-mid central  frontal area | VPA, AZD, CZP, PB, CLB, TPM, LTG, LEV, ESM | VPA, AZD, CZP | None | Refractory | Not clear (Epilepsy Phenome/Genome Project) | ^[26]^ |
| 1 day/F | Status epilepticus, EOEE | - | - | p. A1511S | Missense | UN | Migration of rhythmic  left predominant 2–4 Hz  bi-occipital activity to right;  5–6 Hz theta activity;  L > R posterior quadrant  slowing | LEV, STP, CBZ, VPA, TPM, RFM, VGB, ZNS | EV, STP | None | Refractory | Not clear (Epilepsy Phenome/Genome Project) | ^[26]^ |
| UN | Unclassified epilepsy | - | - | p.V1396M | Missense | GOF | Bi-frontal, L fronto-central,  and temporal slowing | TPM, LTG, CLB, VPA  LEV, KD | TPM | None | UN | Not clear (Epilepsy Phenome/Genome Project) | ^[26]^ |
| UN | Juvenile myoclonic epilepsy | - | - | p.E735A | Missense | UN | UN | UN | UN | UN | UN | Korea | ^[27]^ |
| UN | Juvenile myoclonic epilepsy | - | - | p. L238fs | Nonsense | LOF | UN | UN | UN | UN | UN | Korea | ^[27]^ |
| UN | Juvenile myoclonic epilepsy | FS | - | p.R1060C | Missense | UN | UN | UN | UN | UN | UN | Korea | ^[27]^ |
| UN | Juvenile myoclonic epilepsy | - | - | p. A1083D | Missense | UN | UN | UN | UN | UN | UN | Korea | ^[27]^ |
| UN | Juvenile myoclonic epilepsy | - | - | p.N390D | Missense | UN | UN | UN | UN | UN | UN | Korea | ^[27]^ |
| 5y/F | Focal seizures | - | Fever | p.R1667P | Missense | GOF and LOF | Right temporal as well as gener-  alized periodic discharges, superimposed on diffuse background slowing. | LEV | LEV | LEV | Death | Canada | ^[28]^ |
| 31m/M | Status epilepticus | - | - | p.R1349Q | Missense | GOF | UN | LEV, LTG, TPM | None | None | Refractory | USA | ^[29]^ |
| 4y/F | EE, absence seizures. | Multifocal, atonic | - | p.S1373L | Missense | UN | Diffuse 3-4 Hz delta slowing and frequent, multifocal 2 - 3.5 Hz epileptiform transients, most prominent in the bi-occipital regions | ZNS, Divalproex sodium, LTG | LTG | None | Controlled | USA | ^[30]^ |
| 12y/M | Absence epilepsy | - | - | p.V1812A | Missense | UN | Not clear | UN | UN | UN | UN | Japan | ^[31]^ |
| 9m/M | Status epilepticus, EIMFS | FS/GTCS/TS/ SE | - | p.E101Q | Missense | UN | Multifocal spikes, generalized paroxysmal fast activity | CBZ, LEV,  PB, TPM | None | None | Refractory | India | ^[32]^ |
| 6y/5m/M | EE | - | - | p.R1351* | Nonsense | LOF | Not clear | UN | UN | UN | UN | Australia | ^[33]^ |
| 3y/F | Absence epilepsy | Focal seizures | Fever | p. E137G | Missense | UN | Typical 2.5–3 Hz spike-wave | VPA, LTG, pyridoxine | VPA, LTG | Pyridoxine | Controlled | Chinese | ^[34]^ |
| 1day/F | Status epilepticus, Atypical Rett syndrome | TC | Fever, excited, agitated, or traveling | p.A710T | Missense | UN | Extremely slow background activity with intermittent focal slowing in frontal regions. | PB, TPM, VPA, LEV, OXC, LCS, CBZ, LTG, CLP, ZNS, VGB, RUF, FELB clobazam, ezogabine, primidone, and VNS | None | None | Refractory | USA | ^[35]^ |
| 3y/M | Absence epilepsy | GTCS | - | p.R1820stop | Nonsense | LOF | 3 Hz spike-waves | CLB, PHT, ACTZ, CBZ | None | None | Controlled | UK | ^[20]^ |

**Abbreviations**: ACTZ; Acetazolamide, BZD; Benzodiazepine, CLP; clonazepam, CLB; clobazam, CBZ, Carbamazepine, DEE; developmental epileptic encephalopathy, d; day, EP; epilepsy, EEOE; early onset epileptic encephalopathy, EEIE; early infantile epileptic encephalopathy, EE; epileptic encephalopathy, ESM; Ethosuximide, FELB; felbamate, FosPHT; Fos-phenytoin, GOF-gain-of-function, GTC; generalized tonic-clonic, KD; ketogenic diet, LOF: loss-of-function, LEV; Levetiracetam, LTG; Lamotrigine, LCS; Lacosamide, LZP; lorazepam, LGS; Lennox-Gastaut syndrome, MDZ; midazolam, M; month(s), M; male, NTZ; nitrazepam, OXC; Oxcarbamazepine, PB; Phenobarbital, PHT; phenytoin, P5P; pyridoxal 5-phosphate, RUF; rufinamide, TPM; Topiramate, TLE; temporal lobe epilepsy, TC; tonic-clonic, UN, unknown, VPA; Valproic acid, VNS, vagus nerve stimulation, VGB; vigabatrin, y; year, and ZNS; Zonisamide.

**References**

[1] LE ROUX M, BARTH M, GUEDEN S, et al. CACNA1A-associated epilepsy: Electroclinical findings and treatment response on seizures in 18 patients. [J]. European journal of paediatric neurology : EJPN : official journal of the European Paediatric Neurology Society, 2021, 33: 75-85.

[2] VERRIELLO L, PAULETTO G, NILO A, et al. Epilepsy and episodic ataxia type 2: family study and review of the literature [J]. Journal of neurology, 2021, 268(11): 4296-302.

[3] LIPMAN A R, FAN X, SHEN Y, et al. Clinical and genetic characterization of CACNA1A-related disease [J]. Clinical genetics, 2022, 102(4): 288-95.

[4] ALEHABIB E, KOKOTOVIĆ T, RANJI-BURACHALOO S, et al. Leu226Trp CACNA1A variant associated with juvenile myoclonic epilepsy with and without intellectual disability. [J]. Clinical neurology and neurosurgery, 2022, 213: 107108.

[5] DAMAJ L, LUPIEN-MEILLEUR A, LORTIE A, et al. CACNA1A haploinsufficiency causes cognitive impairment, autism and epileptic encephalopathy with mild cerebellar symptoms [J]. European journal of human genetics : EJHG, 2015, 23(11): 1505-12.

[6] BOLTE K N, ASSAF M, ZACH T, et al. Two Children with Early-Onset Strokes and Intractable Epilepsy, Both with CACNA1A Mutations. [Z]. Child neurology open. 2022: 2329048X221094977.10.1177/2329048X221094977

[7] HOMMERSOM M P, VAN PROOIJE T H, PENNINGS M, et al. The complexities of CACNA1A in clinical neurogenetics [J]. Journal of neurology, 2022, 269(6): 3094-108.

[8] LI X-L, LI Z-J, LIANG X-Y, et al. CACNA1A Mutations Associated With Epilepsies and Their Molecular Sub-Regional Implications. [J]. Frontiers in molecular neuroscience, 2022, 15: 860662.

[9] NIU Y, GONG P, JIAO X, et al. Genetic and phenotypic spectrum of Chinese patients with epilepsy and photosensitivity. [J]. Frontiers in neurology, 2022, 13: 907228.

[10] GUDENKAUF F J, AZAMIAN M S, HUNTER J V, et al. A novel CACNA1A variant in a child with early stroke and intractable epilepsy. [J]. Molecular genetics & genomic medicine, 2020, 8: e1383.

[11] NIU X, YANG Y, CHEN Y, et al. Genotype-phenotype correlation of CACNA1A variants in children with epilepsy [J]. Developmental medicine and child neurology, 2022, 64(1): 105-11.

[12] ZANGALADZE A, ASADI-POOYA A A, ASHKENAZI A, et al. Sporadic hemiplegic migraine and epilepsy associated with CACNA1A gene mutation [J]. Epilepsy & behavior : E&B, 2010, 17(2): 293-5.

[13] KRYGIER M, ZAWADZKA M, SAWICKA A, et al. Reflex seizures in rare monogenic epilepsies [J]. Seizure, 2022, 97: 32-4.

[14] JIANG X, RAJU P K, D'AVANZO N, et al. Both gain-of-function and loss-of-function de novo CACNA1A mutations cause severe developmental epileptic encephalopathies in the spectrum of Lennox-Gastaut syndrome [J]. Epilepsia, 2019, 60(9): 1881-94.

[15] RAJAKULENDRAN S, GRAVES T D, LABRUM R W, et al. Genetic and functional characterisation of the P/Q calcium channel in episodic ataxia with epilepsy. [J]. The Journal of physiology, 2010, 588: 1905-13.

[16] HAYASHIDA T, SAITO Y, ISHII A, et al. CACNA1A-related early-onset encephalopathy with myoclonic epilepsy: A case report. [J]. Brain & development, 2018, 40: 130-3.

[17] YAMAZAKI S, IKENO K, ABE T, et al. Hemiconvulsion-hemiplegia-epilepsy syndrome associated with CACNA1A S218L mutation [J]. Pediatric neurology, 2011, 45(3): 193-6.

[18] STUBBERUD A, O'CONNOR E, TRONVIK E, et al. R1352Q CACNA1A Variant in a Patient with Sporadic Hemiplegic Migraine, Ataxia, Seizures and Cerebral Oedema: A Case Report. [Z]. Case reports in neurology. 2021: 123-30.10.1159/000512275

[19] REINSON K, ÕIGLANE-SHLIK E, TALVIK I, et al. Biallelic CACNA1A mutations cause early onset epileptic encephalopathy with progressive cerebral, cerebellar, and optic nerve atrophy [J]. American journal of medical genetics Part A, 2016, 170(8): 2173-6.

[20] IMBRICI P, JAFFE S L, EUNSON L H, et al. Dysfunction of the brain calcium channel CaV2.1 in absence epilepsy and episodic ataxia [J]. Brain : a journal of neurology, 2004, 127(Pt 12): 2682-92.

[21] ANGELINI C, VAN GILS J, BIGOURDAN A, et al. Major intra-familial phenotypic heterogeneity and incomplete penetrance due to a CACNA1A pathogenic variant. [J]. European journal of medical genetics, 2019, 62: 103530.

[22] KORS E E, MELBERG A, VANMOLKOT K R J, et al. Childhood epilepsy, familial hemiplegic migraine, cerebellar ataxia, and a new CACNA1A mutation. [J]. Neurology, 2004, 63: 1136-7.

[23] ZHANG L, WEN Y, ZHANG Q, et al. CACNA1A Gene Variants in Eight Chinese Patients With a Wide Range of Phenotypes [J]. Frontiers in pediatrics, 2020, 8: 577544.

[24] LV Y, WANG Z, LIU C, et al. Identification of a novel CACNA1A mutation in a Chinese family with autosomal recessive progressive myoclonic epilepsy. [J]. Neuropsychiatric disease and treatment, 2017, 13: 2631-6.

[25] STENDEL C, D'ADAMO M C, WIESSNER M, et al. Association of A Novel Splice Site Mutation in P/Q-Type Calcium Channels with Childhood Epilepsy and Late-Onset Slowly Progressive Non-Episodic Cerebellar Ataxia [J]. International journal of molecular sciences, 2020, 21(11).

[26] De Novo Mutations in SLC1A2 and CACNA1A Are Important Causes of Epileptic Encephalopathies. [J]. American journal of human genetics, 2016, 99: 287-98.

[27] LEE C G, LEE J, LEE M. Multi-gene panel testing in Korean patients with common genetic generalized epilepsy syndromes. [J]. PloS one, 2018, 13: e0199321.

[28] GAUQUELIN L, HAWKINS C, TAM E W Y, et al. Pearls & Oy-sters: Fatal brain edema is a rare complication of severe CACNA1A-related disorder. [J]. Neurology, 2020, 94: 631-4.

[29] HO C Y, LOVE H L, SOKOL D K, et al. Longitudinal MRI brain findings in the R1349Q pathogenic variant of CACNA1A [J]. Radiology case reports, 2021, 16(6): 1276-9.

[30] BYERS H M, BEATTY C W, HAHN S H, et al. Dramatic Response After Lamotrigine in a Patient With Epileptic Encephalopathy and a De NovoCACNA1A Variant. [J]. Pediatric neurology, 2016, 60: 79-82.

[31] YAMAMOTO T, IMAIZUMI T, YAMAMOTO-SHIMOJIMA K, et al. Genomic backgrounds of Japanese patients with undiagnosed neurodevelopmental disorders [J]. Brain & development, 2019, 41(9): 776-82.

[32] MITTA N, MENON R N, MCTAGUE A, et al. Genotype-phenotype correlates of infantile-onset developmental & epileptic encephalopathy syndromes in South India: A single centre experience. [J]. Epilepsy research, 2020, 166: 106398.

[33] KOTHUR K, HOLMAN K, FARNSWORTH E, et al. Diagnostic yield of targeted massively parallel sequencing in children with epileptic encephalopathy [J]. Seizure, 2018, 59: 132-40.

[34] DU X, CHEN Y, ZHAO Y, et al. Dramatic response to pyridoxine in a girl with absence epilepsy with ataxia caused by a de novo CACNA1A mutation. [J]. Seizure, 2017, 45: 189-91.

[35] EPPERSON M V, HAWS M E, STANDRIDGE S M, et al. An Atypical Rett Syndrome Phenotype Due to a Novel Missense Mutation in CACNA1A [J]. Journal of child neurology, 2018, 33(4): 286-9.
