# Supplementary material for: The genotype–phenotype correlations of the CACNA1A-related neurodevelopmental disorders: a small case series and literature reviews
Source: Front Mol Neurosci. 2023 Jul 24;16:1222321. doi: 10.3389/fnmol.2023.1222321 (PMC10406136; doi:10.3389/fnmol.2023.1222321)
Supplement: Supplementary file 3 [file Table_3.docx]

**Supplementary Table 3** The comparison of some clinical factors between carriers of the GOF and LOF variants.

| **Variable** | **GOF** | **LOF** | **Total** | **P value** |
| --- | --- | --- | --- | --- |
| **Status epilepticus** |  |  |  |  |
| Yes | 31 (72.1%) | 6 (15.8%) | 37 (45.7%) | 0.000 |
| No | 12 (27.9%) | 32 (84.2%) | 44 (54.3%) |  |
|  |  |  |  |  |
| **Absence seizures** |  |  |  |  |
| Yes | 1 (2.4%) | 18 (47.4%) | 19 (24.1%) | 0.000 |
| No | 40 (97.6%) | 20 (52.6%) | 60 (75.9) |  |
|  |  |  |  |  |
| **Myoclonic epilepsy** |  |  |  |  |
| Yes | 8 (18.6%) | 2 (5.3%) | 10 (12.3%) | 0.094 |
| No | 35 (81.4%) | 36 (94.7%) | 71 (87.7%) |  |
|  |  |  |  |  |
| **Febrile seizures /fever triggered** |  |  |  |  |
| Yes | 9 (20.9%) | 8 (21.6%) | 17 (21.3%) | 1.000 |
| No | 34 (79.1%) | 29 (78.4%) | 63 (78.8%) |  |
|  |  |  |  |  |
| **Treatment outcome** |  |  |  |  |
| Refractory seizures | 25 (59.5%) | 19 (59.4%) | 44 (59.5%) | 1.000 |
| Controlled seizures | 17 (40.5%) | 13 (40.6%) | 30 (40.5%) |  |

**Abbreviations:** GOF; gain-of-function, LOF; loss-of-function.
