# Supplementary material for: The genotype–phenotype correlations of the CACNA1A-related neurodevelopmental disorders: a small case series and literature reviews
Source: Front Mol Neurosci. 2023 Jul 24;16:1222321. doi: 10.3389/fnmol.2023.1222321 (PMC10406136; doi:10.3389/fnmol.2023.1222321)
Supplement: Supplementary file 4 [file Table_4.docx]

**Supplementary Table 4** Determinants of different epileptic manifestations

| **Variable** | **Status epilepticus** | **No status epilepticus** | **Z value** | **P value** |
| --- | --- | --- | --- | --- |
| **Type of mutation** |  |  |  |  |
| Missense | 53/56 (94.6%) | 48/71 (67.6%) |  | 0.000 |
| Nonsense | 3/56 (5.4%) | 23/71 (32.4%) | NAD |  |
| **Position** |  |  |  |  |
| S3 location | 2 (3.6%) | 5 (13%) |  |  |
| S4 location | 7 (22.5%) | 3 (7.9%) |  |  |
| S5 location | 22 (39.2%) | 2 (5.3%) | -4.838 | 0.000 |
| S6 location | 15 (26.8%) | 1 (2.6%) |  |  |
| Extracellular | 2 (3.6%) | 5 (13.2%) |  |  |
| Cytoplasmic | 8 (14.3%) | 22 (57.9%) |  |  |
| **Variants (position)** |  |  |  |  |
| p.A710T (S6) | 2 (6.1%) | 0 (0.0%) |  |  |
| p.I711M (S6) | 2 (6.1%) | 0 (0.0%) |  |  |
| p.A712T (S6) | 2 (6.1%) | 0 (0.0%) | NAD | NAD |
| p.A713T (S6) | 6 (18.2%) | 4 (100%) |  |  |
| p.V1392M (S5) | 8 (24.24%) | 0 (0.0%) |  |  |
| p.V1393M (S5) | 7 (21.2%) | 0 (0.0%) |  |  |
| p.R1348Q/ p.R1349Q (S4) | 3 (9.1%) | 0 (0.0%) |  |  |
| p.Y62C (cytoplasmic) | 3 (9.1%) | 0 (0.0%) |  |  |
| **Position** | **Absence seizures** | **No absence seizures** |  |  |
| S1 location | 0 (0.0%) | 2 (3.4%) |  |  |
| S2 location | 3 (14.3%) | 2 (3.4%) |  |  |
| S3 location | 1 (4.8%) | 5 (8.6%) | -0.144 | 0.885 |
| S4 location | 3 (14.3%) | 7 (12.1%) |  |  |
| S5 location | 0 (0.0%) | 11 (19%) |  |  |
| S6 location | 2 (9.5%) | 6 (10.34%) |  |  |
| Extracellular | 5 (23.8%) | 5 (8.6%) |  |  |
| Cytoplasmic | 7 (33.3%) | 20 (34.5%) |  |  |
|  |  |  |  |  |
| **Position** | **Myoclonic seizures** | **No myoclonic seizures** |  |  |
| S1 location | 1 (9.1%) | 1 (1.5%) |  |  |
| S2 location | 0 (0.0%) | 3 (4.5%) |  |  |
| S3 location | 0 (0.0%) | 7 (10.4%) | -1.280 | 0.201 |
| S4 location | 1 (9.1%) | 7 (10.4%) |  |  |
| S5 location | 1 (9.1%) | 10 (14.9%) |  |  |
| S6 location | 1 (9.1%) | 7 (10.4%) |  |  |
| Extracellular | 0 (0.0%) | 8 (11.9%) |  |  |
| Cytoplasmic | 7 (63.6%) | 24 (35.8%) |  |  |

**Abbreviations**: S; membrane-spanning helix, NAD; not applicable.
