# Supplementary material for: The genotype–phenotype correlations of the CACNA1A-related neurodevelopmental disorders: a small case series and literature reviews
Source: Front Mol Neurosci. 2023 Jul 24;16:1222321. doi: 10.3389/fnmol.2023.1222321 (PMC10406136; doi:10.3389/fnmol.2023.1222321)
Supplement: Supplementary file 5 [file Table_5.docx]

**Supplementary Table 5** Determinants of seizure outcome for both GOF and LOF groups

| **Variable** | **Controlled seizures** | **Refractory seizures** | **Total** | **P value** |
| --- | --- | --- | --- | --- |
| TPM | 6/36 (16.7%) | 28/54 (51.9%) | 34/90 (37.8%) | 0.001 |
| PB | 0/36 (0.0%) | 13/54 (24.1%) | 13/90 (14.4%) | 0.001 |
| LEV | 11/36 (30.6%) | 40/54 (74.1%) | 51/90 (56.7%) | 0.000 |
| PHT | 2/35 (5.7%) | 9/54 (16.7%) | 11/90 (12.2%) | 0.189 |
| VPA | 21/36 (58.3%) | 28/54 (51.9%) | 49/90 (54.4%) | 0.666 |
| LTG | 6/36 (16.7%) | 18/54 (33.3%) | 24/90 (26.7%) | 0.093 |
| CBZ | 8/36 (22.2%) | 17/54 (31.5%) | 25/90 (27.8%) | 0.472 |
| ESM | 3/36 (8.3%) | 5/54 (9.3%) | 8/90 (8.9%) | 1.000 |

**Abbreviations:** CBZ; Carbamazepine, ESM; Ethosuximide, GOF; Gain-of-function, LEV; Levetiracetam, LTG; Lamotrigine, LOF; Loss-of-function, PB; Phenobarbital, PHT; phenytoin, TPM; Topiramate and VPA; Sodium Valproate.
