# Supplementary material for: The genotype–phenotype correlations of the CACNA1A-related neurodevelopmental disorders: a small case series and literature reviews
Source: Front Mol Neurosci. 2023 Jul 24;16:1222321. doi: 10.3389/fnmol.2023.1222321 (PMC10406136; doi:10.3389/fnmol.2023.1222321)
Supplement: Supplementary file 6 [file Table_6.docx]

**Supplementary Table 6** Therapies used and their outcome in GOF group

| **Variable** | **Refractory seizures** | **Controlled seizure** | **Total** | **P value** |
| --- | --- | --- | --- | --- |
| ACTZ | 1/16 (6.3%) | 0/8 (0.0%) | 1/24 (4.2%) | 1.000 |
| TPM | 8/16 (50%) | 1/7 (12.5 % | 9/24 (37.5%) | 0.178 |
| PB | 5/16 (31.3%) | 0/8 (0.0%) | 5/24 (20.8%) | 0.130 |
| LEV | 12/16 (75%) | 2/8 (25%) | 14/24 (58.3%) | 0.032 |
| VPA | 8/16 (50%) | 3/8 (37.5%) | 11/24 (45.8%) | 0.679 |
| LTG | 7/16 (43.8%) | 0/8 (0.0%) | 7/24 (29.2%) | 0.054 |
| CBZ | 7/16 (43.8%) | 3/8 (37.5%) | 10/24 (41.7%) | 1.000 |
| ESM | 1/16 (6.3%) | 0/8 (0.0%) | 1/24 (4.2%) | 1.000 |
| PTH | 3/16 (18.8%) | 1/8 (12.5%) | 4/24 (16.7%) | 1.000 |

**Abbreviations:** ACTZ; acetazolamide, CBZ; Carbamazepine, ESM; Ethosuximide, GOF; Gain-of-function, LEV; Levetiracetam, LTG; Lamotrigine, PB; Phenobarbital, PHT; phenytoin, TPM; Topiramate and VPA; Sodium Valproate.
