# Supplementary material for: The genotype–phenotype correlations of the CACNA1A-related neurodevelopmental disorders: a small case series and literature reviews
Source: Front Mol Neurosci. 2023 Jul 24;16:1222321. doi: 10.3389/fnmol.2023.1222321 (PMC10406136; doi:10.3389/fnmol.2023.1222321)
Supplement: Supplementary file 7 [file Table_7.docx]

**Supplementary Table 7** Therapies used and their outcome in LOF group

| **Variable** | **Refractory seizures** | **Controlled seizures** | **Total** | **P value** |
| --- | --- | --- | --- | --- |
| ACTZ | 2/15 (13.3%) | 1/13 (7.1%) | 3/29 (10.3%) | 1.000 |
| TPM | 7/15 (46.7%) | 2/14 (14.3%) | 9/29 (31%) | 0.109 |
| PB | 2/15 (13.3%) | 0/14 (0.0%) | 2/29 (6.9%) | 0.483 |
| LEV | 5/15 (33.3%) | 5/14 (35.7%) | 10/29 (10/29) | 1.000 |
| VPA | 7/15 (46.7%) | 10/14 (71.4%) | 17/29 (58.6%) | 0.264 |
| LTG | 4/15 (26.7%) | 1/14 (7.1%) | 5/29 (17.2%) | 0.330 |
| CBZ | 3/15 (20%) | 2/14 (14.3%) | 5/29 (17.2%) | 1.000 |
| ESM | 3/15 (20%) | 3/14 (21.4%) | 6/29 (20.7%) | 1.000 |
| PHT | 2/15 (13.3%) | 1/14 (7.1%) | 3/29 (10.3%) | 1.000 |

**Abbreviations:** ACTZ; acetazolamide, CBZ; Carbamazepine, ESM; Ethosuximide, LOF; loss-of-function, LEV; Levetiracetam, LTG; Lamotrigine, PB; Phenobarbital, PHT; phenytoin, TPM; Topiramate and VPA; Sodium Valproate.
