# Supplementary material for: The genotype–phenotype correlations of the CACNA1A-related neurodevelopmental disorders: a small case series and literature reviews
Source: Front Mol Neurosci. 2023 Jul 24;16:1222321. doi: 10.3389/fnmol.2023.1222321 (PMC10406136; doi:10.3389/fnmol.2023.1222321)
Supplement: Supplementary file 8 [file Table_8.docx]

**Supplementary Table 8 *CACNA1A* GDD/ID related variants in 187 patients**

| **Onset age/sex** | **Syndrome/**  **phenotype** | **Other clinical features/ organs affected** | **Nucleotide or protein change** | **Type of mutation** | **Altered protein function** | **MRI results** | **Severity of the GDD/ID** | **Race/Publication Country** | **Reference** |
| --- | --- | --- | --- | --- | --- | --- | --- | --- | --- |
| 5y11m/M | ID, and ataxia | Slight nystagmus, pectus excavatum, and hypotonia bilaterally | p.G701R | Missense | LOF | Cerebellar atrophy and large left ventricular temporal horn | Profound ID | Chinese | Our hospital |
| 5y11m/F | ID, EP, and ataxia | Absence seizures, dizziness, loss of consciousness and memory, paroxysmal limb weakness, and walk instability. | p. R279C | Missense | LOF | Normal | Mild ID | Chinese | Our hospital |
| 10y/M | ID, EP, and ataxia | Fainting attacks and loss of consciousness | p. D1644N | Missense | GOF | Cerebellar atrophy, mild enlargement of right ventricle, temporal horn, and occipital cistern. | Profound ID | Chinese | Our hospital |
| 1y20d/M | ID, EP, and ataxia | - | p.Y62C | Missense | GOF | Abnormal signal in the right hippocampus. The left hippocampus was slightly flat and the temporal horn of bilateral ventricles was enlarged | Severe ID | Chinese | Our hospital |
| UN/F | ID and ataxia | - | p. R1664Q | Missense | LOF | Progressive cerebellar atrophy | Mild ID | Chinese | Our hospital |
| UN | ID, EP, ataxia, and headache | Nystagmus | p.I712V | Missense | UN | Progressive cerebellar and cerebral atrophy | Severe ID | Canada | ^[1]^ |
| UN/M | ID and EP | Gaze-evoked nystagmus | p.V1695L | Missense | UN | Progressive cerebellar atrophy, cerebral atrophy | Severe ID | French and  Belgian | ^[2]^ |
| UN/M | ID, EP, ASD | Hypotonia, cerebellar ataxia, pyramidal signs, ASD; gaze-evoked nystagmus | p.A713T | Missense | GOF | Progressive cerebellar atrophy, cerebral atrophy, hippocampal sclerosis | Severe ID | French and  Belgian | ^[2]^ |
| UN/F | ID, EP | Hypotonia, pyramidal signs, dystonia; PTU, strabismus | p.A713T | Missense | GOF | Normal | Severe ID | French and  Belgian | ^[2]^ |
|  | ID, EP | Hypotonia, central deafness; abnormal visual contact | p.D669A | Missense | UN | Normal | Severe ID | French and  Belgian | ^[2]^ |
| UN/F | ID, EP, cerebellar ataxia, ASD | - | p.R1664* | Nonsense | LOF | Normal | Moderate ID | French and  Belgian | ^[2]^ |
| UN/F | ID, EP, cerebellar ataxia | - | p.R583* | Nonsense | LOF | Normal | Moderate ID | French and  Belgian | ^[2]^ |
| UN/F | ID, EP, cerebellar ataxia | Cerebellar ataxia, gaze-evoked nystagmus | p.R583* | Nonsense | LOF | Normal | Moderate ID | French and  Belgian | ^[2]^ |
| UN/M | ID, EP | - | p.R583* | Nonsense | LOF | Progressive cerebellar atrophy (vermis) | Moderate ID | French and  Belgian | ^[2]^ |
| UN/M | ID, EP | - | p.G939Qfs*128 | Nonsense | LOF | Progressive cerebellar atrophy  (vermis) | Severe ID | French and  Belgian | ^[2]^ |
| UN/F | ID, EP | Hypotonia, cerebellar ataxia, ASD; hypermetropia | p.R279C | Missense | LOF | Progressive cerebellar atrophy  (vermis), widening of  percerebral spaces | Severe ID | French and  Belgian | ^[2]^ |
| UN/F | ID, EP | Hypotonia, cerebellar ataxia; PTU, oculo-motor apraxia | p.G361E | Missense | UN | Progressive cerebellar atrophy | Severe ID | French and  Belgian | ^[2]^ |
| UN/M | ID, EP | Cerebellar ataxia, pyramidal signs, ASD; gaze-evoked nystagmus | p.A713T | Missense | GOF | Progressive cerebellar atrophy,  cerebral atrophy | Severe ID | French and  Belgian | ^[2]^ |
| UN/M | ID, EP | - | p.R1349Q | Missense | GOF | Progressive cerebellar atrophy, left lenticulo-caudal and occipital stroke sequels | Severe ID | French and  Belgian | ^[2]^ |
| UN/M | ID, EP | Cerebellar ataxia; gaze-  evoked nystagmus | p.S616T | Missense | UN | Progressive cerebellar atrophy  (vermis) | Moderate ID | French and  Belgian | ^[2]^ |
| UN/M | ID, EP | Cerebellar ataxia, nystagmus,  strabismus | p.V1393M | Missense | UN | Cerebral atrophy | Severe ID | French and  Belgian | ^[2]^ |
| UN/F | ID, EP, ASD | Pyramidal signs, dystonia, | p.A713T | Missense | GOF | Normal | Severe ID | French and  Belgian | ^[2]^ |
| UN/F | ID, EP | - | p.E1425* | Nonsense | LOF | Normal | Moderate ID | French and  Belgian | ^[2]^ |
| 2y/F | GDD, EP | Recurring status epilepticus, and cerebral infarctions related to seizure episodes. | p.A713T | Missense | GOF | Recurrent ischemic cerebrovascular accident | Severe ID | USA | ^[3]^ |
| 17m/F | GDD, EP | recurrent refractory seizures, and recurring episodes of hemiplegia related to | p.C1369R | Missense | UN | Periventricular leukoencephalopathy | Severe ID | USA | ^[3]^ |
| 11y/F | ID, EP | - | p.R68L | Missense | UN | Normal | ID | Chinese | ^[4]^ |
| 3m/F | ID, EP | - | p.G1322E | Missense | UN | Normal | ID | Chinese | ^[4]^ |
| 1.5y/M | ID, EP | - | p.S1798L | Missense | GOF | Normal | ID | Chinese | ^[4]^ |
| 4y/F | ID, EP | - | p.L1692Q | Missense | GOF | Progressive diffuse cerebral atrophy | Severe ID | USA | ^[5]^ |
| UN/M | ID, ataxia | - | p.G1105S | Missense | LOF | UN | ID | UK | ^[6]^ |
| UN/M | ASD, ID, ataxia | - | p.E921D | Missense | LOF | UN | ID | UK | ^[6]^ |
| UN/M | ASD, ID, ataxia | - | p.E921D | Missense | LOF | UN | ID | UK | ^[6]^ |
| 5y/F | ID, familial hemiplegic migraine | - | p.S218L | Missense | UN | UN | ID | Japanese | ^[7]^ |
| 1y/M | ID, EP | - | p.V1808L | Missense | UN | Progressive white matter cerebral atrophy and thin corpus callosum | Severe ID | Japanese | ^[8]^ |
| UN | EIEE | Not clear | p.V1812A | Missense | UN | UN | Mild ID | Japanese | ^[9]^ |
| UN | ID and episodic ataxia | Psychotic symptoms | p.R822Pfs*246 | Frameshift | LOF | UN | ID | Italy | ^[10]^ |
| UN | ID and episodic ataxia | None | c.3698+1G>A | Aberrant splicing | UN | UN | ID | Italy | ^[10]^ |
| UN | ID and episodic ataxia | None | c.3698+1G>A | Aberrant splicing | UN | UN | ID | Italy | ^[10]^ |
| UN | ID, cerebellar ataxia, and migraine | Depression | p.V581L | Missense | UN | UN | ID | Germany | ^[11]^ |
| UN | GDD, congenital ataxia, and hemiplegic migraine with cerebral edema. | None | p.F1502del | Deletion | GOF | Progressive cerebellar and cerebral atrophy | GDD | , Switzerland | ^[12]^ |
| UN | ID, EP, prolonged attacks of migraine with hemiplegia, ataxia, and coma | None | p.Y1385C | Missense | UN | Unchanged cerebellar and cerebral atrophy | ID | France | ^[13]^ |
| UN | ID and ataxia | Telangiectasia, nystagmus, and exophthalmos | p.G682W | Missense | UN | Normal | ID | Japanese | ^[14]^ |
| UN | GDD and ataxia | Nystagmus | p.T666M | Missense | UN | Congenital cerebellar atrophy | Moderate GDD | UK | ^[15]^ |
| UN | GDD | Bilateral esotropia | p.P1353L | Missense | LOF | Normal | GDD | Minnesota | ^[16]^ |
| UN | EE | None | p.R1351* | Nonsense | LOF | Unknown | GDD | Australia | ^[17]^ |
| UN | EOEE | None | p.R279C | Missense | LOF | Cerebellar atrophy | GDD | France | ^[18]^ |
| UN | GDD, early-onset cerebellar ataxia and dysarthria | Nystagmus and moderate conductive deafness | p.R279C | Missense | LOF | Cerebellar atrophy | Mild ID | France | ^[18]^ |
| UN | ID, early-onset cerebellar ataxia and dysarthria | Nystagmus | p.R279C | Missense | LOF | Progressive cerebellar atrophy | Mild ID | France | ^[18]^ |
| UN | GDD and EOEE | Blind and mild dysmorphic features | p.R158Tfs*6 | Frameshift | LOF | Progressive cerebral, cerebellar, and optic nerve atrophy | Profound GDD | Estonia | ^[19]^ |
| UN | GDD, EOEE, migraine, vertigo attacks | Blind and psychiatric symptoms | p.R158Tfs*6 | Frameshift | LOF | Progressive cerebral, cerebellar, and optic nerve atrophy | Profound GDD | Estonia | ^[19]^ |
| UN | ID, EP, and adult-onset ataxia | Myoclonus | p.A713T | Missense | GOF | UN | ID | Germany | ^[20]^ |
| UN | GDD, progressive cerebellar ataxia, coma, and hemiplegia | Paroxysmal tonic upgaze | p.R1349Q | Missense | GOF | Cerebellar atrophy | GDD | Australia | ^[21]^ |
| UN | GDD, progressive cerebellar ataxia, coma, hemiplegia | Paroxysmal tonic upgaze | p.R1349Q | Missense | GOF | Cerebellar atrophy | GDD | Australia | ^[21]^ |
| UN | ID and migraine | Paroxysmal tonic upgaze | p.R1666H | Missense | UN | Normal | ID | Australia | ^[21]^ |
| UN | GDD, progressive cerebellar ataxia, and ADHD | Dysmetric saccades and esotropic trabismus | p.R583Q | Missense | UN | Normal | GDD | Australia | ^[21]^ |
| UN | GDD and cerebellar ataxia | Dysmetric saccades | p.D1337Y | Missense | UN | Cerebellar vermis  atrophy | GDD | Australia | ^[21]^ |
| UN | GDD, episodic ataxia, and migraine | Paroxysmal tonic upgaze | p.G297R | Missense | UN | Normal | GDD | Australia | ^[21]^ |
| UN | GDD, episodic ataxia, and migraine | Alternating, non-accommodative esotropic strabismus | p.S218L | Missense | UN | Unknown | GDD | Australia | ^[21]^ |
| UN | ID, static cerebellar signs, hypotonia, and coordination difficulties | Benign paroxysmal tonic upgaze | p.Q681Rfs*Xaa17 | Frameshift | LOF | Normal | Moderate ID | France | ^[22]^ |
| UN | ID and episodic ataxia | Benign paroxysmal tonic upgaze | p.Q681Rfs*Xaa17 | Frameshift | LOF | Progressive cerebellar vermis atrophy | Mild ID | France | ^[22]^ |
| UN | ID and episodic ataxia | None | p.T1458M | Missense | UN | Progressive cerebellar vermis atrophy | Moderate ID | France | ^[22]^ |
| UN | ID and episodic ataxia | Nystagmus | p.G677R | Missense | UN | Progressive cerebellar vermis atrophy | Mild ID | France | ^[22]^ |
| UN | ID, congenital ataxia, and dyskinesia | None | p.R1350G | Missense | UN | Progressive cerebellar vermis atrophy | ID | Israel | ^[23]^ |
| UN | ID and comatose episodes | Blindness and nystagmus | p.S218L | Missense | UN | Progressive cerebellar and cerebral atrophy | ID | Spain | ^[24]^ |
| UN | ID and comatose episodes | None | p.S218L | Missense | UN | Progressive cerebellar atrophy | ID | Netherlands | ^[25]^ |
| UN | ID, comatose episodes, and hemiplegia | Nystagmus | p.T666M | Missense | UN | UN | ID | Japanese | ^[26]^ |
| UN | ID, comatose episodes, and hemiplegia | Nystagmus | p.G5361T | Missense | UN | Hemispheric edema | ID | Germany | ^[27]^ |
| UN | ID, comatose episodes, and hemiplegia | Nystagmus | p.G5361T | Missense | UN | Normal | ID | Germany | ^[27]^ |
| UN | ID, EP, ataxia and nystagmus | Nystagmus | p.R1278* | Nonsense | LOF | Hippocampal asymmetry | Severe ID | Canada | ^[28]^ |
| UN | ID, ataxia and nystagmus | Nystagmus | p.R957fs* | Frameshift | LOF | UN | Mild ID | Canada | ^[28]^ |
| UN | ID, EP, ataxia and ADHD | Nystagmus | p.R957fs* | Frameshift | LOF | UN | Mild ID | Canada | ^[28]^ |
| UN | ID, EP, ataxia and ADHD | Nystagmus | c.868+5Gly4A | Splice-site | LOF | UN | Mild ID | Canada | ^[28]^ |
| UN | ID, ataxia, and ADHD | Nystagmus | Del19p13.13 | Deletion | LOF | UN | Mild ID | Canada | ^[28]^ |
| UN | ID, ataxia and ADHD | Nystagmus | Del19p13.13 | Deletion | LOF | Normal | Mild-moderate ID | Canada | ^[28]^ |
| UN | ID and ataxia | None | c.959G>A | Splice site | UN | UN | Low IQ | Austria | ^[29]^ |
| UN | ID and ataxia | Schizophrenia | c.3102+2T>C | Splice site | UN | UN | Low IQ | Austria | ^[29]^ |
| UN | ID and ataxia | Anxiety disorder | c.3603dup | Duplication | UN | UN | Low IQ | Austria | ^[29]^ |
| UN | ID and ataxia | Depression | p.R198Q | Missense | UN | UN | Low IQ | Austria | ^[29]^ |
| UN | ID and ataxia | None | p.S218L | Missense | GOF | UN | Low IQ | Austria | ^[29]^ |
| UN | ID and ataxia | None | p.G540R | Missense | UN | UN | Low IQ | Austria | ^[29]^ |
| UN | ID and ataxia | None | p.W670C | Missense | UN | UN | Low IQ | Austria | ^[29]^ |
| UN | ID and ataxia | None | p.R1668W | Missense | UN | UN | Low IQ | Austria | ^[29]^ |
| UN | Epileptic encephalopathy and ID | None | p.G230V | Missense | LOF | Normal | Moderate ID | North America | ^[30]^ |
| UN | ID, ataxia, and LGS | Tremors | p.A713T | Missense | GOF | Normal | Severe ID | North America | ^[30]^ |
| UN | ID, EE, and ataxia | Nystagmus | p.I1357S | Missense | LOF | Progressive cerebellar atrophy | Severe ID | North America | ^[30]^ |
| UN | GDD, EE, and ataxia | Optic nerve glioma and tremors | p.V1396M and  p.G2314S | Missense | GOF | Normal | Moderate GDD | North America | ^[30]^ |
| UN | ID, migraine and ataxia | Nystagmus | c.1063dupG | Duplication | UN | UN | Severe ID | France | ^[31]^ |
| UN | GDD and ataxia | Abnormal behaviors, hyperreflexia and eye movement disorder | p.R1664Q | Missense | LOF | Normal | GDD | USA | ^[32]^ |
| UN | GDD and ataxia | Esotropia, hyperopia, a tented upper lip and prominent jaw | p.R1673P | Missense | GOF but later it was revealed to be LOF | Progressive cerebellar atrophy, thin corpus callosum; mild delay in deep white matter myelination | GDD | USA, Aurora | ^[32, 33]^ |
| UN | GDD and ataxia | Difficulty sleeping, sensory processing disorder, strabismus, myopia, and astigmatism | p.R1664Q | Missense | LOF | Thin corpus callosum | GDD | USA | ^[32]^ |
| UN | GDD and ataxia | ADHD, hyporeflexic, alternating strabismus, and esotropia | p.R1664Q | Missense | LOF | Progressive atrophy of cerebellar vermis | GDD | USA | ^[32]^ |
| UN | GDD and ataxia | Aggression and ocular apraxia | p.R1664Q | Missense | LOF | Progressive atrophy of cerebellar vermis | GDD | USA | ^[32]^ |
| UN | GDD, EP, and ASD | None | p.P2312_Q2313ins | Insertion | UN | Partial agenesis of the corpus callosum | GDD | Canada | ^[34]^ |
| UN | Atypical Rett Syndrome | Tremors and ataxia | p.A710T | Missense | UN | Mild hypo-myelination | ID | USA | ^[35]^ |
| UN | ID, EP, and ataxia | None | p.S1799L | Missense | UN | Progressive cerebellar  atrophy | ID | Japan | ^[36]^ |
| UN | ID, and ADHD | Strabismus, ataxia | p.Q1154* | Nonsense | LOF | Normal | Mild to moderate ID | France | ^[37]^ |
| UN | EIMFS | Strabismus, nystagmus, ataxia, tremor, and athetosis. | p.E101Q | Missense | UN | Normal | Severe ID | Not clear (Epilepsy Phenome/Genome Project) | ^[38]^ |
| UN | EOEE | Strabismus, nystagmus, ataxia, tremor, and athetosis. | p.S218L | Missense | GOF | Hypoxic lesion | Severe ID | Not clear (Epilepsy Phenome/Genome Project) | ^[38]^ |
| UN | EOEE | Strabismus, nystagmus, ataxia, tremor, and athetosis. | p.A713T | Missense | GOF | Bimesial temporal lobe increased T2 signal | Severe ID | Not clear (Epilepsy Phenome/Genome Project) | ^[38]^ |
| UN | EOEE | Strabismus, nystagmus, ataxia, tremor, and athetosis. | p.A713T | Missense | GOF | Normal | Severe ID | Not clear (Epilepsy Phenome/Genome Project) | ^[38]^ |
| UN | EOEE | Strabismus, nystagmus, ataxia, tremor, and athetosis. | p. A1511S | Missense | UN | Normal | Moderate ID | Not clear (Epilepsy Phenome/Genome Project) | ^[38]^ |
| UN | ID and ataxia | None | c.4503-4505delCTT | Deletion | LOF | Progressive cerebellar atrophy | Severe ID | Spain | ^[39]^ |
| Om/M | GDD | Congenital cerebellar ataxia and paroxysmal tonic upward gaze | p.R1348Q | Missense | UN | None | GDD | Israel | ^[40]^ |
| 0.2m/F | GDD | Congenital cerebellar ataxia and paroxysmal tonic upward gaze | p.C272Y | Missense | UN | None | GDD | Israel | ^[40]^ |
| 1m/M | GDD | Congenital cerebellar ataxia | p.R1663Q | Missense | UN | None | GDD | Israel | ^[41]^ |
| 10m/F | GDD | Congenital cerebellar ataxia, paroxysmal tonic upward gaze, epilepsy, and febrile seizures | p.E1267K | Missense | UN | None | GDD | Israel | ^[41]^ |
| 11m/F | GDD | Congenital cerebellar ataxia, paroxysmal tonic upward gaze, and epilepsy | p.A1807T | Missense | UN | None | GDD | Israel | ^[41]^ |
| 0.2m/F | GDD | Congenital cerebellar ataxia and paroxysmal tonic upward gaze | p.V1350L | Missense | UN | None | GDD | Israel | ^[41]^ |
| 12m/M | GDD | Congenital cerebellar ataxia and language delay | p.I1342T | Missense | UN | None | GDD | Israel | ^[41]^ |
| 22m/F | GDD | Suspected congenital cerebellar ataxia | p.S218L | Missense | GOF | None | GDD | Israel | ^[41]^ |
| 7m/M | GDD | Congenital cerebellar ataxia and paroxysmal tonic upward gaze, epilepsy, and oculomotor apraxia. | p.V1396M | Missense | UN | None | GDD | Israel | ^[41]^ |
| 3m/M | GDD | Congenital cerebellar ataxia, paroxysmal tonic upward gaze | p.R1352Q | Missense | UN | None | GDD | Israel | ^[41]^ |
| 12m/M | GDD | Congenital cerebellar ataxia, paroxysmal tonic upward gaze, and language delay | p.A1511D | Missense | UN | None | GDD | Israel | ^[41]^ |
| 12m/F | GDD | Congenital cerebellar ataxia, paroxysmal tonic upward gaze, and persistent dystonia | p.R1669Q | Missense | UN | None | GDD | Israel | ^[41]^ |
| 9m/F | GDD | Congenital cerebellar ataxia, paroxysmal tonic upward gaze, and febrile convulsions | p.V1396M | Missense | UN | None | GDD | Israel | ^[41]^ |
| 12m/F | GDD | Congenital cerebellar ataxia, and paroxysmal tonic upward gaze | p.Y1387C | Missense | UN | None | GDD | Spain | ^[42]^ |
| 12m/M | GDD | Congenital cerebellar ataxia, paroxysmal tonic upward gaze, paroxysmal torticollis, and hemi-dystonia | p.F1502del | Deletion | GOF | None | GDD | Spain | ^[39]^ |
| 12m/M | GDD | Paroxysmal tonic upward gaze, paroxysmal vertigo, and episodic ataxia | rs786200963 | Splice site | UN | None | GDD | Israel | ^[41]^ |
| 11m/F | GDD | None | p.Q681Rfs*17 | Frameshift | LOF | None | GDD | France | ^[43]^ |
| 8m/F | GDD | Congenital cerebellar ataxia, paroxysmal tonic upward gaze, and paroxysmal vertigo | p.Q681Rfs*17 | Frameshift | LOF | None | GDD | France | ^[43]^ |
| 9m/F | GDD | Congenital cerebellar ataxia, and episodic ataxia | c.3825+1G>A | Splice-site | UN | None | GDD | France | ^[22]^ |
| 10m/M | GDD | Congenital cerebellar ataxia, and hemi-dystonia | p.C1417W | Missense | UN | None | GDD | France | ^[22]^ |
| 12m/M | GDD | Paroxysmal tonic upward gaze | p.G677R | Missense | UN | None | GDD | France | ^[22]^ |
| 1m/F | GDD | Paroxysmal tonic upward gaze and suspected congenital cerebellar ataxia | p.D668A | Missense | UN | None | GDD | Israel | ^[41]^ |
| 1m/F | GDD | Suspected congenital cerebellar ataxia | p.A712T | Missense | UN | None | GDD | Not clear (Epilepsy Phenome/Genome Project) | ^[44]^ |
| 11m/F | GDD | Paroxysmal tonic upward gaze and suspected congenital cerebellar ataxia | p.S1663N | Missense | UN | None | GDD | Israel | ^[41]^ |
| 0.5m/M | GDD | Paroxysmal tonic upward gaze, paroxysmal torticollis and paroxysmal vertigo | p.D302N | Missense | UN | None | GDD | Germany | ^[45]^ |
| 3m/F | GDD | Congenital cerebellar ataxia, paroxysmal tonic upward gaze | p.N1189Tfs*5  3 | Frameshift | LOF | None | GDD | Israel | ^[41]^ |
| 2m/F | GDD | Congenital cerebellar ataxia, paroxysmal tonic upward gaze | p.R1756Q | Missense | UN | None | GDD | Israel | ^[41]^ |
| 0m/F | GDD | Paroxysmal tonic upward gaze | p. R1362W | Missense | UN | None | GDD | Israel | ^[41]^ |
| Died within 3-6m | GDD | Hypotonia, mild facial  dysmorphic features, encephalopathy, and seizures were born | p.R932* | Nonsense | LOF | UN | Died | Spain | ^[46]^ |
| Died within 3-6m | GDD | Hypotonia, mild facial dysmorphic features, encephalopathy, and seizures were born | p.R932* | Nonsense | LOF | UN | Died | Spain | ^[46]^ |
| Died at the age of 5y | ID and fatal brain edema | Hypotonia, eye movement abnormalities, ocular motor apraxia, cerebellar ataxia, dysarthria, and generalized dystonia | p.R1667P | Missense | UN | Isolated progressive cerebellar vermis atrophy | Died | Canada | ^[47]^ |
|  | GDD | Hemiplegic migraine, cerebellar atrophy | p.Y1384C | Missense | LOF | Progressive early onset cerebellar atrophy | GDD | Canada | ^[48]^ |
| 11y/F | ID | Focal seizures, migraine, cerebellar ataxia, nystagmus, and motor delay | p.F363S | Missense | UN | UN | ID | France | ^[49]^ |
| 12y/M | ID | Febrile seizures | p.R1349Q | Missense | GOF | UN | Mild ID | France | ^[49]^ |
| 5y/M | ID | Loss of consciousness, motor delay, and migraine headache | p.Y1384C | Missense | LOF | UN | Mild ID | France | ^[49]^ |
| 2y/F | ID | Loss of consciousness, motor delay, and focal seizures | p.F1506S | Missense | UN | UN | ID | France | ^[49]^ |
| 6m/M | ID | Cerebellar ataxia, migraine, epilepsy, coma, brain edema. | p.R1352Q | Missense | UN | UN | Mild ID | UK | ^[50]^ |
| UN | ID | Hemiplegic migraine, episodic ataxia, strabismus, dysarthria, nystagmus, and febrile seizures | p.R1352Q | Missense | UN | UN | ID | Chinese | ^[51]^ |
| UN | ID | Hemiplegic migraine, episodic ataxia, strabismus, f febrile seizures, and non-progressing ataxia; febrile seizures and status epilepticus | p.R1352Q | Missense | UN | UN | ID | Chinese | ^[51]^ |
| UN | ID | Migraine headache and non-progressing ataxia, febrile seizures and status epilepticus, | p.G701V | Missense | UN | UN | ID | Chinese | ^[51]^ |
| UN | GDD | Non-progressing ataxia, status tremor, febrile seizures, and status epilepticus | p.A713T | Missense | GOF | UN | ID | Chinese | ^[51]^ |
| UN | GDD | Non-progressing ataxia, status tremor, febrile seizures, and status epilepticus | p.A713T | Missense | GOF | UN | ID | Chinese | ^[51]^ |
| UN/F | GDD | Paroxysmal tonic upward gaze | p.R1349* | Nonsense | LOF | UN | GDD | Chinese | ^[52]^ |
| UN/F | GDD | Paroxysmal tonic upward gaze | p.S1472L | Missense | UN | UN | GDD | Chinese | ^[52]^ |
| UN | ID | Epilepsy and cerebellar ataxia | p.K595Sfs*17 | Frameshift | LOF | UN | ID | Netherlands | ^[53]^ |
| UN | ID | Epilepsy, ataxia, nystagmus | p.A952Sfs*115 | Frameshift | LOF | UN | Mild ID | Netherlands | ^[53]^ |
| UN | ID | Ataxia | p.R1352Q |  | UN | UN | Mild ID | Netherlands | ^[53]^ |
| UN | ID | Epilepsy | p.R1661H | Missense | UN | UN | ID | Netherlands | ^[53]^ |
| UN | GDD | Muscle  weak  ness | p.I1709T | Missense | UN | UN | GDD | Netherlands | ^[53]^ |
| UN | ID | None | p.G1755R | Missense | UN | UN | GDD | Netherlands | ^[53]^ |
| UN | ID | None | p.Y339* | Nonsense | LOF | UN | GDD | Netherlands | ^[53]^ |
| UN | ID | None | p.R1780* | Nonsense | LOF | UN | ID | Netherlands | ^[53]^ |
| UN/F | ID | Hypotonia, truncal ataxia, and stereotypes  Strabismus, terminal nystagmus, ASD, ADHD | p.F1394L | Missense | UN | Cerebellar atrophy | Severe ID | Spain | ^[54]^ |
| UN/M | ID | Hypotonia, ataxia, dysarthria, oculomotor apraxia,  nystagmus and abnormal movements | p.R1664Q | Missense | UN | Progressive cerebellar atrophy | Mild ID | Spain | ^[54]^ |
| UN/F | ID | Ataxia, hypotonia, ADHD | p.R1669Q | Missense | UN | Progressive cerebellar atrophy | Mild ID | Spain | ^[54]^ |
| 21y/F | Epilepsy | Abnormality of eye movement, nystagmus, tremor | p.L226W | Missense | UN | Normal | NAD | Austria | ^[55]^ |
| 16y/F | Epilepsy | Abnormality of eye movement, nystagmus, tremor | p.L226W | Missense | UN | Normal | NAD | Austria | ^[55]^ |
| UN/F | GDD and EP | Microcephaly, severe hypotonia, dysmorphic features, growth failure, nystagmus, mild cortical visual impairment, truncal instability, and truncal ataxia | R1667P | Missense | Both GOF and LOF | Pontocerebellar hypoplasia, thinning of the corpus callosum, small cerebellum, brainstem, and pons, delayed myelination in the cerebellum with gliotic white matter changes. | Severe ID | USA | ^[56]^ |
| 3y/F | GDD | High‑grade fever and altered sensorium Hypotonia left hemiparesis, and ataxia presented with incessant crying, vomiting, and decreased responsiveness. | p.Y1383C | Missense | UN | Progressive cerebellar atrophy. | Severe GDD | India | ^[57]^ |
| 6 m/F | Severe EOEE and DEE | Fever, hypotonia, status epilepticus, fixed flexion contractures in her elbows, hips, and knees | p.Q681Rfs*100 and p.E565K | Missense and frameshift | LOF | Progressive cerebellar atrophy | Severe GDD | UK | ^[58]^ |
| 2d//F | EIEE | None | p.A713T | GOF | UN | Normal | ID | Chinese | ^[59]^ |
| 1y 4m /F | DEE | Autistic features | p.V1393M | Missense | UN | Right cerebral atrophy | ID | Chinese | ^[59]^ |
| 8y 6m/F | Absence epilepsy with paroxysmal ataxia | Ataxia | p.Q680Rfs*100 | Frameshift | LOF | Normal | ID | Chinese | ^[59]^ |
| 3m /F | DEE | Hypotonia | p.G1323E | Missense | UN | Left cerebral atrophy, corpus callosum dysplasia | ID | Chinese | ^[59]^ |
| 1d/F | EIEE | Poor swallowing, masticatory weakness, | p.A711T | Missense | UN | Right cerebral atrophy | ID | Chinese | ^[59]^ |
| 1y/F | Epilepsy and GDD | Right facial paralysis | p.Y62C | Missense | GOF | Left cerebral atrophy | ID | Chinese | ^[59]^ |
| 7.5m/F | DEE | – | p.V1393M | Missense | UN | Normal | ID | Chinese | ^[59]^ |
| 1y 6m /M | DEE | Eye squint | p.I759T | Missense | UN | Normal | ID | Chinese | ^[59]^ |
| 1m /F | EIEE | – | p.S1469L | Missense | UN | Normal | ID | Chinese | ^[59]^ |
| 5m/M | EIEE | – | p.V1393M | Missense | UN | Normal | ID | Chinese | ^[59]^ |
| 4m/F | EIEE | – | p.R55S | Missense | UN | Normal | ID | Chinese | ^[59]^ |
| 5m/F | EIEE | Ataxia | p.Q685* | Nonsense | LOF | Normal | ID | Chinese | ^[59]^ |
| 5m/M | EIEE | – | c.6530-1G＞C | Splice site | UN | Normal | ID | Chinese | ^[59]^ |
| 1y 5m/M | DEE | – | p.V1393M | Missense | UN | Progressive left cerebral atrophy | ID | Chinese | ^[59]^ |
| 3y 7m /F | ID and EP | Ataxia | p.G297R | Missense | UN | Normal | ID | Chinese | ^[59]^ |
| 8.5m/F | DEE | Ataxia, tremor | p.V1393M | Missense | UN | Normal | ID | Chinese | ^[59]^ |
| 7y/M | ID and EP | Ataxia | p.W169* | Nonsense | LOF | Normal | ID | Chinese | ^[59]^ |
| 3y 11m/M | ID and EP | – | p.N283S | Missense | UN | Normal | ID | Chinese | ^[59]^ |
| UN/M | ID | - | p.L356P | Missense | UN | UN | ID | Italy | ^[60]^ |
| UN/F | ID and EP | - | p.R1669* | Nonsense | LOF | UN | ID | Italy | ^[60]^ |
| UN/M | ID and EP | - | p.D1320N | Missense | UN | UN | ID | Italy | ^[60]^ |
| 10 y/F | No | Transient dizziness and falling attacks, nausea along with gait instability, Nausea, vomiting, slurred speech, vertigo | c.3836dupA, exon 23, and p.Y1279* | Duplication and nonsense | LOF | Progressive cerebellar atrophy and numerous asymptomatic hyperintense lesions of the cerebral white matter. | ID | Chinese | ^[61]^ |
| UN | ID and EP | UN | p.Q680Rfs*100 | Frameshift | LOF | Normal | UN | Chinese | ^[62]^ |
| 58 y/M | ID and ataxia | Severe generalized ataxia, horizontal gaze-evoked nystagmus, cognitive impairment, and a positive family history of gait difficulties. | p. E995* | Nonsense | LOF | Progressive cerebrum and cerebellum atrophy.  Shoulder girdle atrophy | Mild ID | Germany | ^[63]^ |
| 6 m/M | GDD and EP | Left hemiparesis, epilepsy, and stroke-like episodes, infantile onset of motor delay and nystagmus followed by multiple episodes of intractable seizures, strokes, and hemiplegic migraine throughout childhood | p.R1349Q | Missense | GOF | Hemispheric encephaloma- lacia with mismatched perfusion and angiographic imaging, in addition to progressive cerebellar atrophy. | UN | USA | ^[64]^ |

**Abbreviations**: ADHD; attention deficit hyperactive disorder, ASD; autism spectrum disorder, DEE; developmental and epileptic encephalopathy, EE; epileptic encephalopathy, EP; epilepsy, EIEE; early infantile epileptic encephalopathy, EOEE; early onset epileptic encephalopathy, F; female, GDD; global developmental delay, GOF: gain-of-function, ID; intellectual disability, LGS; Lennox-gastaut syndrome, LOF: loss-of-function, MRI; magnetic resonance imaging, m; month, M; male, NAD; not applicable, UN; unknown.

**References**

[1] GUERIN A A, FEIGENBAUM A, DONNER E J, et al. Stepwise developmental regression associated with novel CACNA1A mutation. [J]. Pediatric neurology, 2008, 39: 363-4.

[2] LE ROUX M, BARTH M, GUEDEN S, et al. CACNA1A-associated epilepsy: Electroclinical findings and treatment response on seizures in 18 patients [J]. European journal of paediatric neurology : EJPN : official journal of the European Paediatric Neurology Society, 2021, 33: 75-85.

[3] BOLTE K N, ASSAF M, ZACH T, et al. Two Children with Early-Onset Strokes and Intractable Epilepsy, Both with CACNA1A Mutations [J]. Child neurology open, 2022, 9: 2329048x221094977.

[4] LI X L, LI Z J, LIANG X Y, et al. CACNA1A Mutations Associated With Epilepsies and Their Molecular Sub-Regional Implications [J]. Frontiers in molecular neuroscience, 2022, 15: 860662.

[5] GUDENKAUF F J, AZAMIAN M S, HUNTER J V, et al. A novel CACNA1A variant in a child with early stroke and intractable epilepsy. [J]. Molecular genetics & genomic medicine, 2020, 8: e1383.

[6] RAJAKULENDRAN S, GRAVES T D, LABRUM R W, et al. Genetic and functional characterisation of the P/Q calcium channel in episodic ataxia with epilepsy [J]. The Journal of physiology, 2010, 588(Pt 11): 1905-13.

[7] YAMAZAKI S, IKENO K, ABE T, et al. Hemiconvulsion-hemiplegia-epilepsy syndrome associated with CACNA1A S218L mutation [J]. Pediatric neurology, 2011, 45(3): 193-6.

[8] HAYASHIDA T, SAITO Y, ISHII A, et al. CACNA1A-related early-onset encephalopathy with myoclonic epilepsy: A case report [J]. Brain & development, 2018, 40(2): 130-3.

[9] YAMAMOTO T, IMAIZUMI T, YAMAMOTO-SHIMOJIMA K, et al. Genomic backgrounds of Japanese patients with undiagnosed neurodevelopmental disorders. [J]. Brain & development, 2019, 41: 776-82.

[10] MANTUANO E, ROMANO S, VENEZIANO L, et al. Identification of novel and recurrent CACNA1A gene mutations in fifteen patients with episodic ataxia type 2. [J]. Journal of the neurological sciences, 2010, 291: 30-6.

[11] FREILINGER T, ACKL N, EBERT A, et al. A novel mutation in CACNA1A associated with hemiplegic migraine, cerebellar dysfunction and late-onset cognitive decline [J]. Journal of the neurological sciences, 2011, 300(1-2): 160-3.

[12] GARCÍA SEGARRA N, GAUTSCHI I, MITTAZ-CRETTOL L, et al. Congenital ataxia and hemiplegic migraine with cerebral edema associated with a novel gain of function mutation in the calcium channel CACNA1A. [J]. Journal of the neurological sciences, 2014, 342: 69-78.

[13] VAHEDI K, DENIER C, DUCROS A, et al. CACNA1A gene de novo mutation causing hemiplegic migraine, coma, and cerebellar atrophy [J]. Neurology, 2000, 55(7): 1040-2.

[14] KASHIMADA A, HASEGAWA S, NOMURA T, et al. Genetic analysis of undiagnosed ataxia-telangiectasia-like disorders. [J]. Brain & development, 2019, 41: 150-7.

[15] NAIK S, POHL K, MALIK M, et al. Early-onset cerebellar atrophy associated with mutation in the CACNA1A gene [J]. Pediatric neurology, 2011, 45(5): 328-30.

[16] WEYHRAUCH D L, YE D, BOCZEK N J, et al. Whole Exome Sequencing and Heterologous Cellular Electrophysiology Studies Elucidate a Novel Loss-of-Function Mutation in the CACNA1A-Encoded Neuronal P/Q-Type Calcium Channel in a Child With Congenital Hypotonia and Developmental Delay. [J]. Pediatric neurology, 2016, 55: 46-51.

[17] KOTHUR K, HOLMAN K, FARNSWORTH E, et al. Diagnostic yield of targeted massively parallel sequencing in children with epileptic encephalopathy. [J]. Seizure, 2018, 59: 132-40.

[18] ANGELINI C, VAN GILS J, BIGOURDAN A, et al. Major intra-familial phenotypic heterogeneity and incomplete penetrance due to a CACNA1A pathogenic variant. [J]. European journal of medical genetics, 2019, 62: 103530.

[19] REINSON K, ÕIGLANE-SHLIK E, TALVIK I, et al. Biallelic CACNA1A mutations cause early onset epileptic encephalopathy with progressive cerebral, cerebellar, and optic nerve atrophy [J]. American journal of medical genetics Part A, 2016, 170(8): 2173-6.

[20] BALCK A, HANSSEN H, HELLENBROICH Y, et al. Adult-onset ataxia or developmental disorder with seizures: two sides of missense changes in CACNA1A [J]. Journal of neurology, 2017, 264(7): 1520-2.

[21] TANTSIS E M, GILL D, GRIFFITHS L, et al. Eye movement disorders are an early manifestation of CACNA1A mutations in children [J]. Developmental medicine and child neurology, 2016, 58(6): 639-44.

[22] HUMBERTCLAUDE V, RIANT F, KRAMS B, et al. Cognitive impairment in children with CACNA1A mutations [J]. Developmental medicine and child neurology, 2020, 62(3): 330-7.

[23] BLUMKIN L, MICHELSON M, LESHINSKY-SILVER E, et al. Congenital ataxia, mental retardation, and dyskinesia associated with a novel CACNA1A mutation [J]. Journal of child neurology, 2010, 25(7): 892-7.

[24] FITZSIMONS R B, WOLFENDEN W H. Migraine coma. Meningitic migraine with cerebral oedema associated with a new form of autosomal dominant cerebellar ataxia. [J]. Brain : a journal of neurology, 1985, 108 ( Pt 3: 555-77.

[25] KORS E E, TERWINDT G M, VERMEULEN F L, et al. Delayed cerebral edema and fatal coma after minor head trauma: role of the CACNA1A calcium channel subunit gene and relationship with familial hemiplegic migraine [J]. Annals of neurology, 2001, 49(6): 753-60.

[26] WADA T, KOBAYASHI N, TAKAHASHI Y, et al. Wide clinical variability in a family with a CACNA1A T666m mutation: hemiplegic migraine, coma, and progressive ataxia [J]. Pediatric neurology, 2002, 26(1): 47-50.

[27] DE VRIES B, STAM A H, BEKER F, et al. CACNA1A mutation linking hemiplegic migraine and alternating hemiplegia of childhood [J]. Cephalalgia : an international journal of headache, 2008, 28(8): 887-91.

[28] DAMAJ L, LUPIEN-MEILLEUR A, LORTIE A, et al. CACNA1A haploinsufficiency causes cognitive impairment, autism and epileptic encephalopathy with mild cerebellar symptoms [J]. European journal of human genetics : EJHG, 2015, 23(11): 1505-12.

[29] INDELICATO E, NACHBAUER W, KARNER E, et al. The neuropsychiatric phenotype in CACNA1A mutations: a retrospective single center study and review of the literature [J]. European journal of neurology, 2019, 26(1): 66-e7.

[30] JIANG X, RAJU P K, D'AVANZO N, et al. Both gain-of-function and loss-of-function de novo CACNA1A mutations cause severe developmental epileptic encephalopathies in the spectrum of Lennox-Gastaut syndrome. [J]. Epilepsia, 2019, 60: 1881-94.

[31] JUNG J, TESTARD H, TOURNIER-LASSERVE E, et al. Phenotypic variability of episodic ataxia type 2 mutations: a family study. [J]. European neurology, 2010, 64: 114-6.

[32] LUO X, ROSENFELD J A, YAMAMOTO S, et al. Clinically severe CACNA1A alleles affect synaptic function and neurodegeneration differentially. [J]. PLoS genetics, 2017, 13: e1006905.

[33] TYAGI S, BENDRICK T R, FILIPOVA D, et al. A mutation in Ca(V)2.1 linked to a severe neurodevelopmental disorder impairs channel gating. [J]. The Journal of general physiology, 2019, 151: 850-9.

[34] MELOCHE J, BRUNET V, GAGNON P-A, et al. Exome sequencing study of partial agenesis of the corpus callosum in men with developmental delay, epilepsy, and microcephaly. [J]. Molecular genetics & genomic medicine, 2020, 8: e992.

[35] EPPERSON M V, HAWS M E, STANDRIDGE S M, et al. An Atypical Rett Syndrome Phenotype Due to a Novel Missense Mutation in CACNA1A [J]. Journal of child neurology, 2018, 33(4): 286-9.

[36] OHBA C, OSAKA H, IAI M, et al. Diagnostic utility of whole exome sequencing in patients showing cerebellar and/or vermis atrophy in childhood. [J]. Neurogenetics, 2013, 14: 225-32.

[37] BERTHOLON P, CHABRIER S, RIANT F, et al. Episodic ataxia type 2: unusual aspects in clinical and genetic presentation. Special emphasis in childhood. [J]. Journal of neurology, neurosurgery, and psychiatry, 2009, 80: 1289-92.

[38] De Novo Mutations in SLC1A2 and CACNA1A Are Important Causes of Epileptic Encephalopathies. [J]. American journal of human genetics, 2016, 99: 287-98.

[39] BAHAMONDE M I, SERRA S A, DRECHSEL O, et al. A Single Amino Acid Deletion (ΔF1502) in the S6 Segment of CaV2.1 Domain III Associated with Congenital Ataxia Increases Channel Activity and Promotes Ca2+ Influx. [J]. PloS one, 2015, 10: e0146035.

[40] BLUMKIN L, LESHINSKY-SILVER E, MICHELSON M, et al. Paroxysmal tonic upward gaze as a presentation of de-novo mutations in CACNA1A. [J]. European journal of paediatric neurology : EJPN : official journal of the European Paediatric Neurology Society, 2015, 19: 292-7.

[41] GUR-HARTMAN T, BERKOWITZ O, YOSOVICH K, et al. Clinical phenotypes of infantile onset CACNA1A-related disorder [J]. European journal of paediatric neurology : EJPN : official journal of the European Paediatric Neurology Society, 2021, 30: 144-54.

[42] CARREÑO O, GARCÍA-SILVA M T, GARCÍA-CAMPOS Ó, et al. Acute striatal necrosis in hemiplegic migraine with de novo CACNA1A mutation [J]. Headache, 2011, 51(10): 1542-6.

[43] HUMBERTCLAUDE V, KRAMS B, NOGUE E, et al. Benign paroxysmal torticollis, benign paroxysmal vertigo, and benign tonic upward gaze are not benign disorders. [J]. Developmental medicine and child neurology, 2018, 60: 1256-63.

[44] ALLEN A S, BERKOVIC S F, COSSETTE P, et al. De novo mutations in epileptic encephalopathies. [J]. Nature, 2013, 501: 217-21.

[45] BÜRK K, KAISER F J, TENNSTEDT S, et al. A novel missense mutation in CACNA1A evaluated by in silico protein modeling is associated with non-episodic spinocerebellar ataxia with slow progression. [J]. European journal of medical genetics, 2014, 57: 207-11.

[46] ARTECHE-LÓPEZ A, ÁLVAREZ-MORA M I, SÁNCHEZ CALVIN M T, et al. Biallelic variants in genes previously associated with dominant inheritance: CACNA1A, RET and SLC20A2. [J]. European journal of human genetics : EJHG, 2021, 29: 1520-6.

[47] GAUQUELIN L, HAWKINS C, TAM E W Y, et al. Pearls & Oy-sters: Fatal brain edema is a rare complication of severe CACNA1A-related disorder. [J]. Neurology, 2020, 94: 631-4.

[48] GANDINI M A, SOUZA I A, FERRON L, et al. The de novo CACNA1A pathogenic variant Y1384C associated with hemiplegic migraine, early onset cerebellar atrophy and developmental delay leads to a loss of Cav2.1 channel function. [J]. Molecular brain, 2021, 14: 27.

[49] RIANT F, DUCROS A, PLOTON C, et al. De novo mutations in ATP1A2 and CACNA1A are frequent in early-onset sporadic hemiplegic migraine. [J]. Neurology, 2010, 75: 967-72.

[50] STUBBERUD A, O'CONNOR E, TRONVIK E, et al. R1352Q CACNA1A Variant in a Patient with Sporadic Hemiplegic Migraine, Ataxia, Seizures and Cerebral Oedema: A Case Report. [Z]. Case reports in neurology. 2021: 123-30.10.1159/000512275

[51] ZHANG L, WEN Y, ZHANG Q, et al. CACNA1A Gene Variants in Eight Chinese Patients With a Wide Range of Phenotypes [J]. Frontiers in pediatrics, 2020, 8: 577544.

[52] ZHANG L-P, JIA Y, WANG Y-P. Identification of Two de novo Variants of CACNA1A in Pediatric Chinese Patients With Paroxysmal Tonic Upgaze. [J]. Frontiers in pediatrics, 2021, 9: 722105.

[53] HOMMERSOM M P, VAN PROOIJE T H, PENNINGS M, et al. The complexities of CACNA1A in clinical neurogenetics [J]. Journal of neurology, 2022, 269(6): 3094-108.

[54] MARTÍNEZ-MONSENY A F, EDO A, CASAS-ALBA D, et al. CACNA1A Mutations Causing Early Onset Ataxia: Profiling Clinical, Dysmorphic and Structural-Functional Findings. [J]. International journal of molecular sciences, 2021, 22.

[55] ALEHABIB E, KOKOTOVIĆ T, RANJI-BURACHALOO S, et al. Leu226Trp CACNA1A variant associated with juvenile myoclonic epilepsy with and without intellectual disability. [J]. Clinical neurology and neurosurgery, 2022, 213: 107108.

[56] GROSSO B J, KRAMER A A, TYAGI S, et al. Complex effects on Ca(V)2.1 channel gating caused by a CACNA1A variant associated with a severe neurodevelopmental disorder. [J]. Scientific reports, 2022, 12: 9186.

[57] GAJAM S, PETERSON R R, MATHEW A A, et al. Sporadic Hemiplegic Migraine with CACNA1A Mutation Masquerading as Acute Meningoencephalitis. [J]. Annals of Indian Academy of Neurology, 2022, 25: 528-9.

[58] WONG-SPRACKLEN V M Y, KOLESNIK A, ECK J, et al. Biallelic CACNA1A variants: Review of literature and report of a child with drug-resistant epilepsy and developmental delay. [Z]. American journal of medical genetics Part A. 2022: 3306-11.10.1002/ajmg.a.62960

[59] NIU X, YANG Y, CHEN Y, et al. Genotype-phenotype correlation of CACNA1A variants in children with epilepsy [J]. Developmental medicine and child neurology, 2022, 64(1): 105-11.

[60] MELLONE S, PURICELLI C, VURCHIO D, et al. The Usefulness of a Targeted Next Generation Sequencing Gene Panel in Providing Molecular Diagnosis to Patients With a Broad Spectrum of Neurodevelopmental Disorders. [J]. Frontiers in genetics, 2022, 13: 875182.

[61] YUAN X, ZHENG Y, GAO F, et al. Case Report: A Novel CACNA1A Mutation Caused Flunarizine-Responsive Type 2 Episodic Ataxia and Hemiplegic Migraine With Abnormal MRI of Cerebral White Matter. [Z]. Frontiers in neurology. 2022: 899813.10.3389/fneur.2022.899813

[62] NIU Y, GONG P, JIAO X, et al. Genetic and phenotypic spectrum of Chinese patients with epilepsy and photosensitivity. [J]. Frontiers in neurology, 2022, 13: 907228.

[63] SAATHOFF Y, BISKUP S, FUNKE C, et al. New Nonsense Variant c.2983G>T; p.Glu995* in the CACNA1A Gene Causes Progressive Autosomal Dominant Ataxia. [Z]. Journal of movement disorders. 2021: 70-4.10.14802/jmd.20082

[64] HO C Y, LOVE H L, SOKOL D K, et al. Longitudinal MRI brain findings in the R1349Q pathogenic variant of CACNA1A [J]. Radiology case reports, 2021, 16(6): 1276-9.
