# Supplementary material for: The genotype–phenotype correlations of the CACNA1A-related neurodevelopmental disorders: a small case series and literature reviews
Source: Front Mol Neurosci. 2023 Jul 24;16:1222321. doi: 10.3389/fnmol.2023.1222321 (PMC10406136; doi:10.3389/fnmol.2023.1222321)
Supplement: Supplementary file 9 [file Table_9.docx]

**Supplementary Table 9** Correlations between GDD/ID severity and the locations of the variants and recurrent variants

| **Variable** | **Mild-moderate ID/GDD** | **Severe-profound ID/GDD** | **Z value** | **P value** |
| --- | --- | --- | --- | --- |
| **Position** |  |  |  |  |
| S1 location | 0 (0.0%) | 1 (2.8%) |  |  |
| S2 location | 0 (0.0%) | 1 (2.8%) |  |  |
| S3 location | 2 (6.5%) | 2 (5.6%) |  |  |
| S4 location | 8 (25.8%) | 4 (11.1%) | -0.103 | 0.918 |
| S5 location | 4 (12.9%) | 5 (13.9%) |  |  |
| S6 location | 1 (3.2%) | 10 (27.8%) |  |  |
| Extracellular | 8 (25.8%) | 2 (5.6%) |  |  |
| Cytoplasmic | 8 (25.8%) | 11 (30.6%) |  |  |
| **Domain** |  |  |  |  |
| Domain I | 1 (11.1%) | 1 (5.6%) |  |  |
| Domain II | 4 (44.4%) | 10 (55.6%) | -0.056 | 0.955 |
| Domain III | 3 (33.3%) | 4 (22.2%) |  |  |
| Domain IV | 1 (11.1%) | 3 (16.7%) |  |  |
| **Variants (position)** |  |  |  |  |
| p.A713T (S6) | 0 (0.0%) | 8 (61.5%) |  |  |
| p. R1664Q/p. R1664* (S3) | 3 (21.4%) | 0 (0.0%) |  |  |
| p.V1393M (S5) | 0 (0.0%) | 1 (7.7%) |  |  |
| p.S218L (cytoplasmic) | 0 (0.0%) | 1 (7.7%) |  |  |
| p.R1352Q (S4) | 2 (14.3%) | 0 (0.0%) | -2.911 | 0.003 |
| p.R1349Q/p.R1349* (S4) | 1 (7.1%) | 1 (7.7%) |  |  |
| p. R279C (extracellular) | 3 (21.4%) | 1 (7.7%) |  |  |
| p.R583* (S4) | 3 (21.4%) | 0 (0.0%) |  |  |
| p.Q681Rfs*Xaa17 (extracellular) | 2 (14.3%) | 1 (7.7%) |  |  |

**Abbreviations**: GDD; global developmental delay, ID; intellectual disability, S; transmembrane helix.
