# Supplementary material for: The genotype–phenotype correlations of the CACNA1A-related neurodevelopmental disorders: a small case series and literature reviews
Source: Front Mol Neurosci. 2023 Jul 24;16:1222321. doi: 10.3389/fnmol.2023.1222321 (PMC10406136; doi:10.3389/fnmol.2023.1222321)
Supplement: Supplementary file 10 [file Table_10.docx]

**Supplementary Table 10** Determinants of different severity of the GDD/ID

| **Variable** | **Mild-moderate ID/GDD** | **Severe-profound ID/GDD** | **Total** | **P value** |
| --- | --- | --- | --- | --- |
| Male | 4/13 (30.8%) | 10/20 (50%) | 14/33 (42.4%) | 0.310 |
| Female | 9/13 (69.2%) | 10/20 (50%) | 19/33 (57.6%) |  |
|  |  |  |  |  |
| Cerebellar atrophy | 12/33 (36.4%) | 22/37 (59.5%) | 34/70 (48.6%) | 0.061 |
| No cerebellar atrophy | 21/33 (63.6%) | 15/37 (40.5%) | 36/70 (51.4%) |  |
|  |  |  |  |  |
| LOF | 18/20 (90%) | 7/20 (35%) | 25/40 (62.5%) | 0.001 |
| GOF | 2/20 (10%) | 13/20 (65%) | 15/40 (37.5%) |  |
|  |  |  |  |  |
| Missense | 18/33 (54.5%) | 28/36 (77.8%) | 46/69 (66.7%) | 0.072 |
| Nonsense | 15/33 (45.5%) | 8/36 (22.2%) | 23/69 (33.3%) |  |

**Abbreviations**: GDD; global developmental delay, GOF; gain-of-function, ID; intellectual disability, LOF; loss-of-function.
