# Supplementary material for: The genotype–phenotype correlations of the CACNA1A-related neurodevelopmental disorders: a small case series and literature reviews
Source: Front Mol Neurosci. 2023 Jul 24;16:1222321. doi: 10.3389/fnmol.2023.1222321 (PMC10406136; doi:10.3389/fnmol.2023.1222321)
Supplement: Supplementary file 11 [file Table_11.docx]

**Supplementary Table 11** *CACNA1A* variants related to ASD in 18 patients

| **Current or publication age/sex** | **Syndrome/**  **phenotype** | **Other clinical features/ organs affected** | **Nucleotide or protein change** | **Type of mutation** | **Altered protein function** | **Variant location** | **Reference** |
| --- | --- | --- | --- | --- | --- | --- | --- |
| 5y11m/M | ASD | ID, non-episodic ataxia, hypotonic | p. G701R | Missense | LOF | S6 | Our hospital |
| 3y2m/M | ASD | EP, ID, non-episodic ataxia, hypotonic | p.Y62C | Missense | GOF | Cytoplasmic | Our hospital |
| 4.4y/M | ASD | EP, ID, non-episodic ataxia, hypotonic | p.V1392M | Missense | GOF | S5 | ^[1]^ |
| 5.8y/F | ASD | EP, ID, hypotonic | p.V1392M | Missense | GOF | S5 | ^[1]^ |
| 7.3y/F | ASD | EP, ID, non-episodic ataxia, hypotonic | p.A712T | Missense | GOF | S6 | ^[1]^ |
| 28.6y/F | ASD | Ataxia | p.R279C | Missense | LOF | Extracellular | ^[1]^ |
| 4.4y/M | ASD | EP, ID, non-episodic ataxia, hypotonic | p.A712T | Missense | GOF | S6 | ^[1]^ |
| 12.9y/M | ASD | EP, ID, non-episodic ataxia, hypotonic | p.R1348Q | Missense | GOF | S4 | ^[1]^ |
| 13.4y/M | ASD | EP, ID, hypotonic | p.I711M | Missense | GOF | S6 | ^[1]^ |
| **Current or publication age/sex** | **Syndrome/**  **phenotype** | **Other clinical features/ organs affected** | **Nucleotide or protein change** | **Type of mutation** | **Altered protein function** | **Variant location** | **Reference** |
| 11.6y/M | ASD | EP, ID, non-episodic ataxia, hypotonic | p.G700E | Missense | GOF | S6 | ^[1]^ |
| 29.2y/M | ASD | ID, EP | p.S1798L | Missense | GOF | S6 | ^[1]^ |
| 13.5y/M | ASD | EP,ID | p.I1707T | Missense | UN | S5 | ^[1]^ |
| 27y/F | ASD | EP, ID, non-episodic ataxia, | p.V1392M | Missense | GOF | S5 | ^[1]^ |
| Child | ASD | EP, GDD | p.P2312_ Q2313ins | A non‐frameshift insertion | UN | Cytoplasmic | ^[2]^ |
| 36m/M | ASD | Episodic ataxia | p.E533K | Missense | LOF | S2 | ^[3, 4]^ |
| 120m/F | ASD | ADHD, episodic ataxia | p.E533K | Missense | LOF | S2 | ^[3, 4]^ |
| 18m | ASD | GDD, ADHD, EP | p.R1278Ter | Deletion | LOF | S2 | ^[5]^ |
| 20m | ASD | GDD, ADHD | p.R1278Ter | Deletion | LOF | S2 | ^[5]^ |

**Abbreviations**: ASD; autism spectrum disorder, ADHD; attention deficit/hyperactive disorder, EP; epilepsy, F; female, GOF; gain of function, ID; intellectual disability, LOF; loss of function, m; month, M; male, UN; unknown., y; year.

References

[1] MARTÍNEZ-MONSENY A F, EDO A, CASAS-ALBA D, et al. CACNA1A Mutations Causing Early Onset Ataxia: Profiling Clinical, Dysmorphic and Structural-Functional Findings [J]. International journal of molecular sciences, 2021, 22(10).

[2] MELOCHE J, BRUNET V, GAGNON P-A, et al. Exome sequencing study of partial agenesis of the corpus callosum in men with developmental delay, epilepsy, and microcephaly. [J]. Molecular genetics & genomic medicine, 2020, 8: e992.

[3] GUR-HARTMAN T, BERKOWITZ O, YOSOVICH K, et al. Clinical phenotypes of infantile onset CACNA1A-related disorder [J]. European journal of paediatric neurology : EJPN : official journal of the European Paediatric Neurology Society, 2021, 30: 144-54.

[4] VILA-PUEYO M, GENÉ G G, FLOTATS-BASTARDES M, et al. A loss-of-function CACNA1A mutation causing benign paroxysmal torticollis of infancy [J]. European journal of paediatric neurology : EJPN : official journal of the European Paediatric Neurology Society, 2014, 18(3): 430-3.

[5] DAMAJ L, LUPIEN-MEILLEUR A, LORTIE A, et al. CACNA1A haploinsufficiency causes cognitive impairment, autism and epileptic encephalopathy with mild cerebellar symptoms [J]. European journal of human genetics : EJHG, 2015, 23(11): 1505-12.
