# Supplementary material for: The genotype–phenotype correlations of the CACNA1A-related neurodevelopmental disorders: a small case series and literature reviews
Source: Front Mol Neurosci. 2023 Jul 24;16:1222321. doi: 10.3389/fnmol.2023.1222321 (PMC10406136; doi:10.3389/fnmol.2023.1222321)
Supplement: Supplementary file 12 [file Data_Sheet_1.doc]

PRISMA Flow Chart

**Screening**

**Included**

**Eligibility**

**Identification**

Records identified through PubMed

(n = 1024)

Additional records identified through other sources
(n =0)

Records after duplicates removed
(n = 1024)

Records screened
(n =1024)

Records excluded
(n = 226)

Full-text articles assessed for eligibility
(n =798)

Full-text articles excluded, with reasons
(n =718)

Studies included in qualitative synthesis
(n = 90)

Studies included in quantitative synthesis (meta-analysis)
(n = 90)
