# Supplementary figures and images for: The genotype–phenotype correlations of the CACNA1A-related neurodevelopmental disorders: a small case series and literature reviews
Source: Front Mol Neurosci. 2023 Jul 24;16:1222321. doi: 10.3389/fnmol.2023.1222321 (PMC10406136; doi:10.3389/fnmol.2023.1222321)

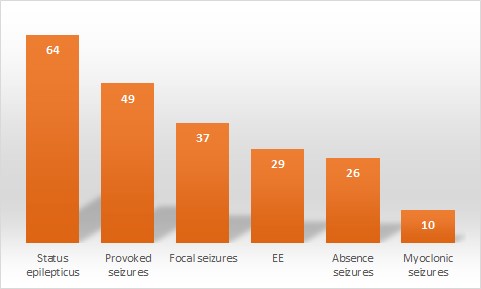

Supplement: Supplementary file 13 [file Image_1.JPEG]

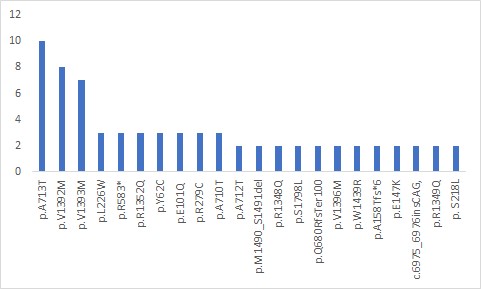

Supplement: Supplementary file 14 [file Image_2.JPEG]

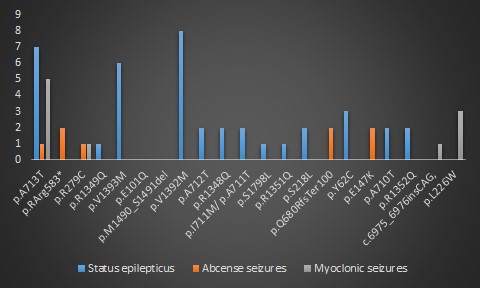

Supplement: Supplementary file 15 [file Image_3.JPEG]

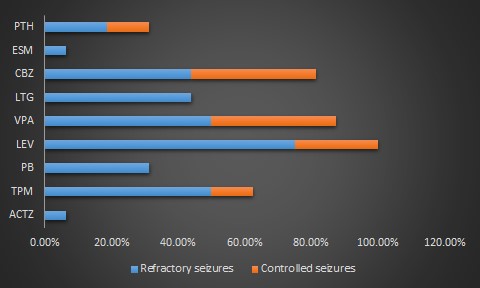

Supplement: Supplementary file 16 [file Image_4.JPEG]

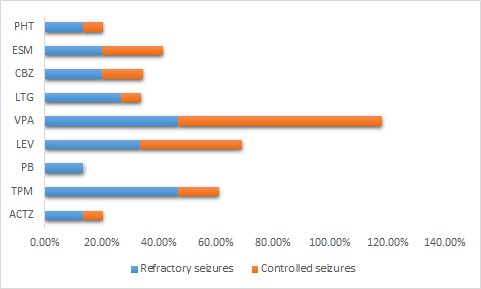

Supplement: Supplementary file 17 [file Image_5.JPEG]

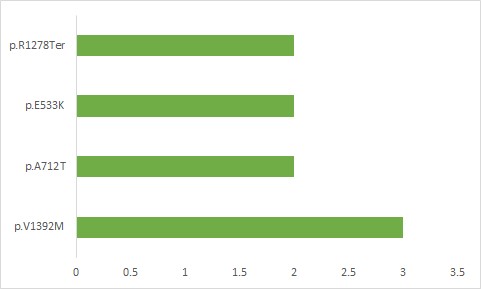

Supplement: Supplementary file 18 [file Image_6.JPEG]

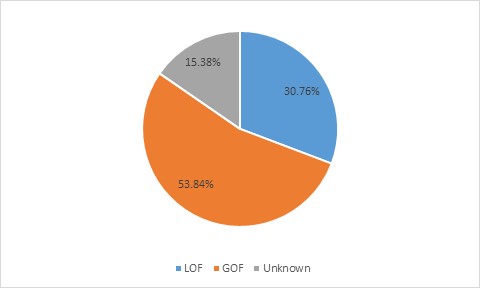

Supplement: Supplementary file 19 [file Image_7.JPEG]
